# ALK5/VEGFR2 dual inhibitor TU2218 alone or in combination with immune checkpoint inhibitors enhances immune-mediated antitumor effects

**DOI:** 10.1007/s00262-024-03777-4

**Published:** 2024-08-06

**Authors:** Nam-Hoon Kim, Jihyun Lee, Seung-Hyun Kim, Seong-Ho Kang, Sowon Bae, Chan-Hee Yu, Jeongmin Seo, Hun-Taek Kim

**Affiliations:** TiumBio Co., Ltd. Seongnam-si, Gyeonggi-do, Republic of Korea

**Keywords:** TGFβ, VEGF, ALK5, VEGFR2, Immuno-oncology

## Abstract

Transforming growth factor β (TGFβ) is present in blood of patients who do not respond to anti-programmed cell death (ligand) 1 [PD-(L)1] treatment, and through synergy with vascular endothelial growth factor (VEGF), it helps to create an environment that promotes tumor immune evasion and immune tolerance. Therefore, simultaneous inhibition of TGFβ/VEGF is more effective than targeting TGFβ alone. In this study, the dual inhibitory mechanism of TU2218 was identified through in vitro analysis mimicking the tumor microenvironment, and its antitumor effects were analyzed using mouse syngeneic tumor models. TU2218 directly restored the activity of damaged cytotoxic T lymphocytes (CTLs) and natural killer cells inhibited by TGFβ and suppressed the activity and viability of regulatory T cells. The inactivation of endothelial cells induced by VEGF stimulation was completely ameliorated by TU2218, an effect not observed with vactosertib, which inhibits only TGFβ signaling. The combination of TU2218 and anti-PD1 therapy had a significantly greater antitumor effect than either drug alone in the poorly immunogenic B16F10 syngeneic tumor model. The mechanism of tumor reduction was confirmed by flow cytometry, which showed upregulated VCAM-1 expression in vascular cells and increased influx of CD8 + CTLs into the tumor. As another strategy, combination of anti-CTLA4 therapy and TU2218 resulted in high complete regression (CR) rates in CT26 and WEHI-164 tumor models. In particular, immunological memory generated by the combination of anti-CTLA4 and TU2218 in the CT26 model prevented the development of tumors after additional tumor cell transplantation, suggesting that the TU2218-based combination has therapeutic potential in immunotherapy.

**Supplementary Information:**

The online version contains supplementary material available at 10.1007/s00262-024-03777-4.

## Introduction

Transforming growth factor β (TGFβ) signaling either inhibits or promotes cancer by regulating various downstream signaling systems. Moreover, TGFβ reduces the proliferation of cancer cells, which indicates that TGFβ hinders cancer progression [1, 2]. However, some studies have reported that TGFβ is overexpressed in cancer cells and involved in the development of cancer owing to its role in epithelial-to-mesenchymal transition [3, 4]. In addition, cancer patients with high TGFβ expression typically have a poor prognosis, and those who do not respond to anti-programmed death ligand 1 [PD-(L)1] therapy have high levels of TGFβ in the blood. Moreover, inhibition of TGFβ signaling in nonclinical experiments increased the efficacy of anti-PD-(L)1 therapy [5–8]. Based on this evidence, TGFβ has been classified as an immuno-anticancer therapeutic target, and the development of clinical TGFβ inhibitors has been actively underway, leading to the accumulation of many clinical studies [9–11]. According to the results of these studies, changes in the tumor microenvironment play important roles in cancer metastasis and growth [12]. Interestingly, TGFβ was found to have a greater effect on stromal and immune cells that surround tumors than on the cancer cells themselves [13]. These findings suggest that TGFβ can cause changes in the tumor microenvironment essential for cancer progression, and, from an immunotherapeutic point of view, this role can be important in the development of cancer therapeutics [13, 14].

As TGFβ exerts its cellular activity by binding to the transmembrane TGFβ type I receptor (also known as activin receptor-like kinase 5 (ALK5)) and type II receptor and forming heterodimer, selective inhibition of ALK5 can result in the blockade of TGFβ signaling cascades [15, 16]. ALK5 is ubiquitously expressed in immune cells, stromal cells, vascular endothelial cells, normal tissues, and tumor cells [17–19]. As a result of its ubiquitous expression, ALK5 alone is insufficient for understanding its clinicopathological role in cancer. Therefore, to clarify the role of ALK5 in the tumor microenvironment, an approach that identifies additional factors that are directly related to the activity of ALK5 and are strongly correlated with the development and severity of the disease was implemented. TGFβ is the most appropriate factor for determining the role of ALK5 [20]. Typically, compared with patients with a low TGFβ-ALK5 signature, cancer patients with an increased TGFβ-ALK5 signature exhibit a noticeable decrease in responsiveness to immune checkpoint inhibitors due to immune evasion and immune exclusion [21, 22]. In other words, from the perspective of immune evasion, ALK5 functions as a suppressor against antigen-presenting cells (APCs), cytotoxic T lymphocytes (CTLs), and natural killer (NK) cells involved in innate immunity and adaptive immunity and, conversely, acts as an activator of regulatory T cells [13]. In addition, from the perspective of immune exclusion, ALK5 acts on stromal cells in the intra- or extratumoral area to promote the formation of the stromal barrier, preventing T cells from infiltrating into the tumor and ultimately promoting refractory cancer and resistance to immunotherapy.

Vascular endothelial growth factor (VEGF) is a major factor that regulates tumor angiogenesis and creates a tumor microenvironment that is favorable for cancer growth by inactivating immune cells [23, 24]. The immunoregulatory property of VEGF can be attributed to the inhibition of dendritic cell differentiation, induction of regulatory T cell (Treg) growth, and inhibition of NK cell and CTL activity [25–27]. Through this immunomodulatory function, VEGF disrupts innate and adaptive immunity in the tumor microenvironment and helps tumors acquire immune evasion and tolerance [28]. In particular, in terms of angiogenesis and immunoregulation, VEGF exerts its effects via vascular endothelial growth factor receptor 2 (VEGFR2) [29]. VEGFR2 is widely expressed in vascular endothelial cells, immune cells, normal tissues, and tumor cells without specific restrictions [30]. In particular, vascular endothelial cells located around tumors are stimulated by various inflammatory cytokines and VEGF secreted from tumor cells and the surrounding immune cells, resulting in endothelial cell inactivation, which reduces the frequency of T cell transmigration from blood vessels into tumors to facilitate immune evasion [27, 31–33]. Inhibition of VEGFR2 has been shown to improve endothelial cell inactivation in tumors and increase the migration of CTLs to tumors, demonstrating that the main determinant of endothelial cell inactivation is suggested to be VEGFR2 in tumor vessel endothelial cells [34]. This hypothesis has been confirmed that combination therapy comprising a tyrosine kinase inhibitor that inhibits VEGFR2 and an anti-PD-(L)1 combination has innovative efficacy in various tumors [35–37], indicating that normalizing vascular immunity by inhibiting the VEGF-VEGFR2 axis is important for improving the efficacy of combination therapy with immune checkpoint inhibitors [33].

Immunosuppression is induced by the synergistic action of TGFβ and VEGF in the tumor microenvironment [38, 39]. In fact, TGFβ and VEGF are overexpressed in advanced cancer [5, 40, 41]. Unfortunately, no current therapeutic agent is capable of effectively inhibiting tumor growth by targeting TGFβ/VEGF. Therefore, a therapeutic agent simultaneously inhibiting TGFβ and VEGF is needed to improve the low response rate to anti-PD-(L)1 monotherapy. Additionally, since ALK5 and VEGFR2 are simultaneously expressed in key cells that are targets of immunotherapy, an efficient treatment strategy based on the mechanism of dual inhibition of ALK5 and VEGFR2 should be identified.

TU2218 is a small-molecule dual inhibitor that targets ALK5 and VEGFR2. Currently, TU2218 monotherapy and its combination with pembrolizumab have undergone phase 1 clinical trials, and phase 2a clinical trials will be conducted in 2024 (NCT05204862, NCT05784688). A pharmacokinetic analysis in a phase 1 clinical trial revealed that the clinical dose of TU2218 can reach to the concentration for inhibiting the phosphorylation of SMAD2 by more than 90%, and can exceed the effective concentration capable of mediating immunotherapeutic effects, and had high safety and tolerability [42]. In this study, we aimed to analyze the nonclinical characteristics of TU2218 related to immuno-oncology by evaluating its dual inhibitory mechanism and antitumor effects and investigated its efficacy in combination with various immune checkpoint inhibitors in a mouse syngeneic tumor model.

## Results

**Fig. 1:**
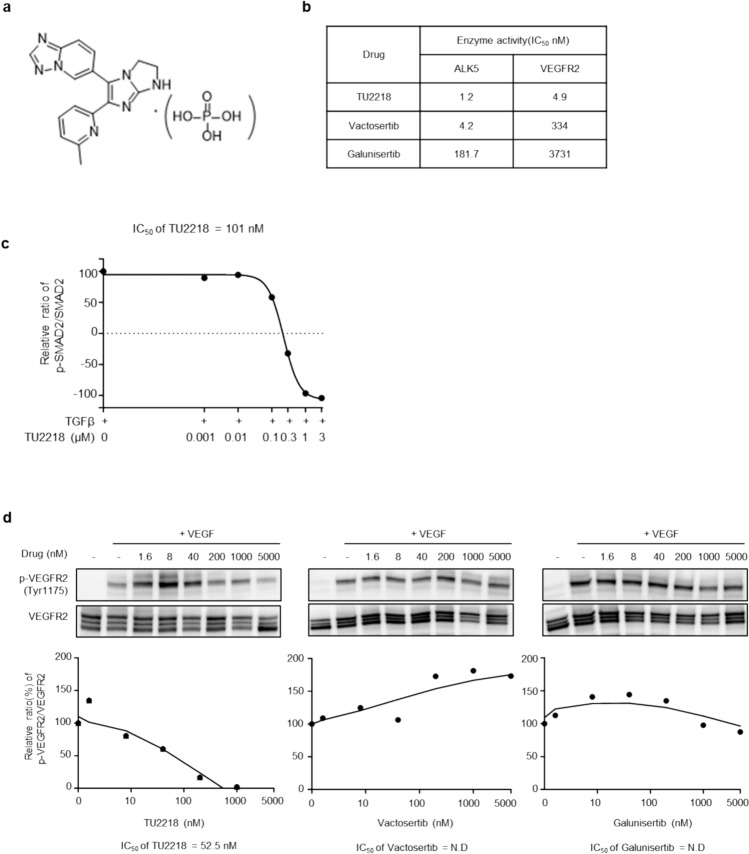
TU2218 is ALK5/VEGFR2 small-molecule inhibitor. **a** Chemical structure of TU2218. **b** Enzyme activity of ALK5 inhibitors. **c** Cellular activity of TU2218 on SMAD2 phosphorylation analyzed in TGFβ-elicited human whole blood by flow cytometry. IC_50_ of SMAD2 phosphorylation were calculated based on mean fluorescence intensity (MFI) of TGFβ-treated cells as a top parameter (100% of SMAD2 phosphorylation) and vehicle as a bottom parameter (0% of SMAD2 phosphorylation). (d) Cellular activity of ALK5 inhibitors on VEGFR2 phosphorylation analyzed in VEGF-stimulated HUVECs

TU2218 is a small-molecule inhibitor that inhibits dual ALK5/VEGFR2 signaling (Fig. 1a). The compounds exerted inhibitory effects on the ALK5 and VEGFR2 proteins at 1.2 nM and 4.9 nM, respectively, which differed from the inhibitory effects of vactosertib and galunisertib, as they inhibited only ALK5 (Fig. 1b). SMAD2 is a representative substrate protein used to measure the activity of ALK5 inhibitors as a sub-signaling protein of ALK5 [43]. TU2218 showed a half maximal inhibitory concentration (IC_50_) of 101 nM in the SMAD2 phosphorylation assay using human whole blood and an IC_50_ of 52.5 nM in the VEGFR2 phosphorylation assay in human umbilical vein endothelial cells (HUVECs) (Fig. 1c, d). Vactosertib and galunisertib were found to have no inhibitory effects on VEGFR2, even under cellular conditions (Fig. 1d).

**Fig. 2:**
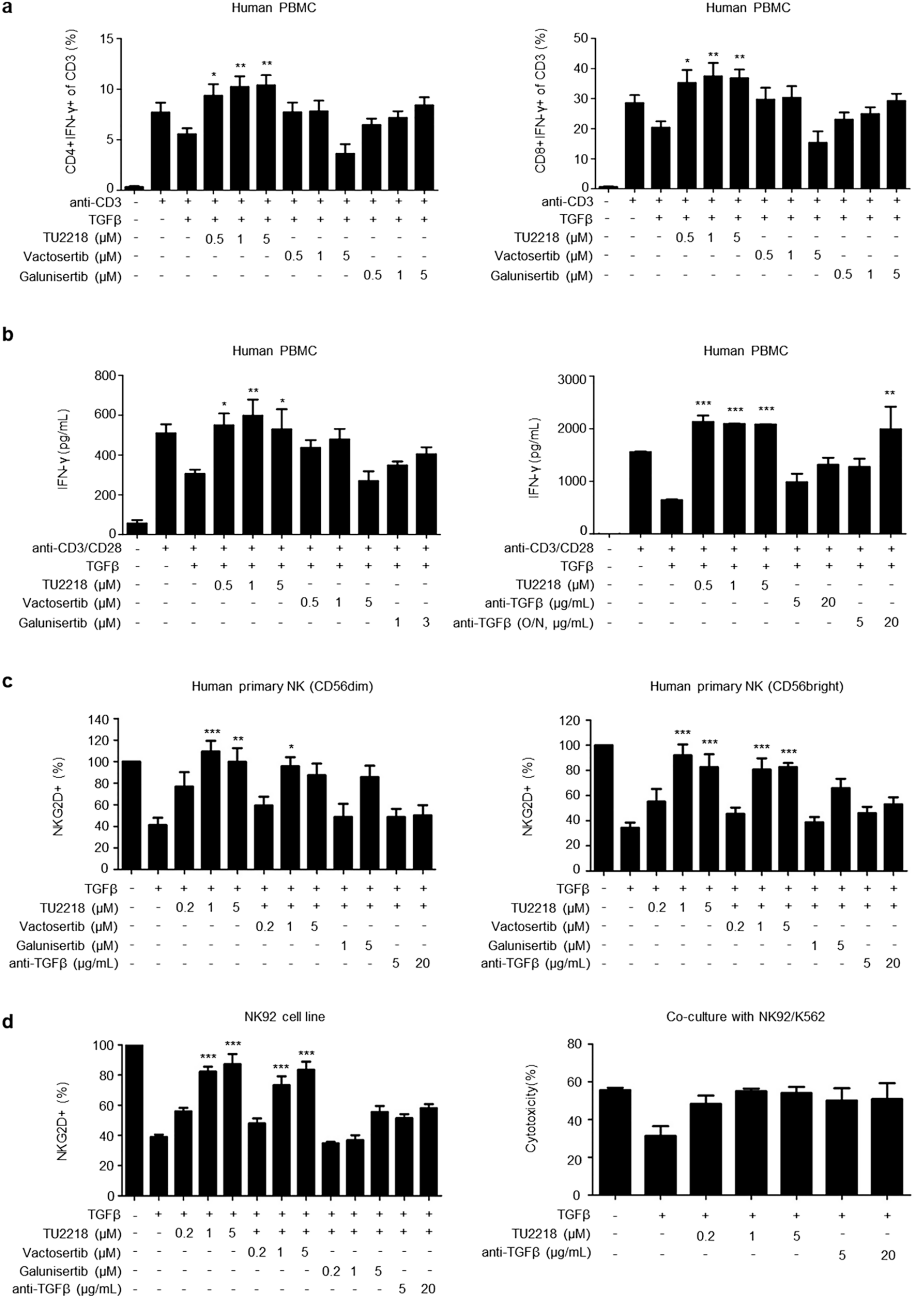

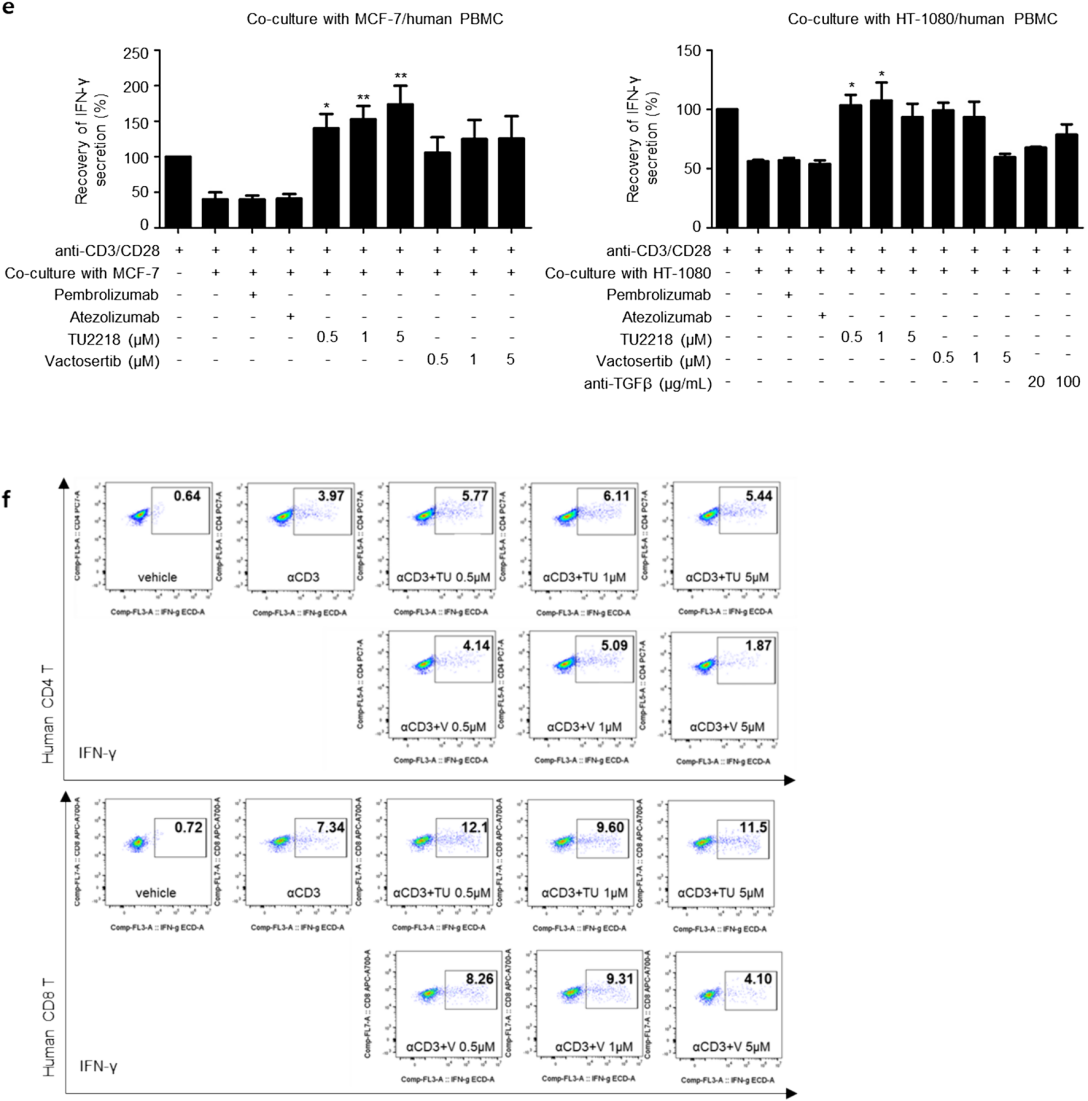
TU2218 inhibits immunosuppression induced by TGFβ or coculture with cancer cells or deficient costimulatory signal. **a** CD4 + IFNγ + or CD8 + IFNγ + of CD3 (%) in PBMCs with indicated treatment condition. Human PBMCs were treated with indicated condition for 24 h and analyzed by flow cytometry. (One-way ANOVA, Dunnett vs. anti-CD3 + TGFβ) **b** IFNγ secretion by indicated treatment condition. The amount of IFNγ in PBMC culture supernatant was measured with ELISA. Human PBMCs were treated with indicated condition for 72 h. **c** The population of NKG2D + cells on CD56^dim^ or CD56^bright^ NK cells in human PBMCs compared to vehicle. NK2GD + cells (%) were assessed by flow cytometry. **d** The population of NKG2D + cells on NK92 cell lines (left) compared to vehicle and cellular cytotoxicity of K562 in NK92/K562 (E/T ratio = 2:1) coculture system (right). **e** Recovery of IFNγ secretion by indicated treatment condition. The amount of IFNγ in PBMC culture supernatant was measured with ELISA and calculated as relative ratio to anti-CD3/CD28 group. **f** Population of IFNγ-producing CD4 or CD8 T lymphocyte subsets in PBMC with indicated treatment condition analyzed by flow cytometry. Noted aCD3 indicates anti-CD3 stimulation, TU indicates TU2218, and V indicates vactosertib. *p ≤ 0.05, **p ≤ 0.01, ***p ≤ 0.001 (One-way ANOVA, Dunnett or Tukey, vs. anti-CD3 + TGFβ or anti-CD3/CD28 + TGFβ or TGFβ)

TGFβ exerts immunosuppressive effects by inhibiting interferon γ (IFNγ) expression in CTLs [44]. In addition, since the effect of TGFβ on CTLs is the major cause of resistance to treatment with immune checkpoint inhibitors, identifying the inhibitory effect of TGFβ on CTLs is important for developing ALK5 inhibitors [7, 44–46]. When human peripheral blood mononuclear cells (PBMCs) stimulated with anti-CD3 antibodies were treated with TGFβ, the reduction in their number was accompanied by decreased IFNγ expression levels in CD4 + and CD8 + CTLs, and this immunosuppressive effect was completely restored by TU2218 treatment (Fig. 2a). The improvement in IFNγ secretion by inhibition of ALK5 activity was statistically significant after TU2218 treatment but not after vactosertib or galunisertib treatment. In addition, similar results were confirmed by analyzing IFNγ secretion in human PBMCs (Fig. 2b). ALK5 inhibition by TU2218 improves the TGFβ-elicited immunosuppression of not only CTLs but also NK cells. TGFβ is involved in lowering NK activity by downregulating the expression of natural killer group 2 member D (NKG2D) receptors, which are activation receptors on the surface of NK cells, and downregulated expression of NKG2D receptors is associated with a poor prognosis in patients with cancer [47, 48]. TU2218 treatment not only significantly upregulated the expression of TGFβ-reduced NKG2D receptors in human primary NK cells (CD56dim and CD56bright) and NK92 cells but also improved the cytotoxic activity of TGFβ-damaged NK cells (Fig. 2c, d, Supplementary figure S1a-g). Moreover, a decrease in IFNγ expression levels is observed in T cells in cocultures of human PBMCs and cancer cells [49]. Such immunosuppression can be explained by immune checkpoint activation resulting from the binding of the PD-L1 protein expressed on the surface of cancer cells with the PD1 protein present in lymphocytes in PBMCs [49]. In addition to direct stimulation by TGFβ, an immune-inactive environment is created by physical contact between cancer cells and immune cells in the tumor microenvironment. IFNγ secretion was significantly decreased when cancer cells such as MCF-7 or HT-1080 cells and PBMCs were cocultured compared to that when T cell receptors (TCRs) were activated in human PBMC monocultures. However, IFNγ secretion, which was reduced in cocultures of PBMCs and cancer cells, was completely restored by TU2218 treatment and improved in a TU2218 concentration-dependent manner (Fig. 2e). Interestingly, IFNγ secretion tended to be improved in response to the TGFβ neutralizing antibody, suggesting that TGFβ was secreted and activated immunosuppression when PBMCs and cancer cells were cocultured. Moreover, overexpression of coinhibitory receptors or defective costimulatory signal in the tumor microenvironment is another immune evasion mechanism [50]. We stimulated only CD3 in human PBMCs without CD28 stimulation, which resulted in the defective activation of costimulatory receptors. Compared with anti-CD3 stimulation alone, TU2218 treatment increased IFNγ secretion in both CD4 + and CD8 + T lymphocytes; this effect was confirmed to be superior to that of vactosertib (Fig. 2f).

**Fig. 3:**
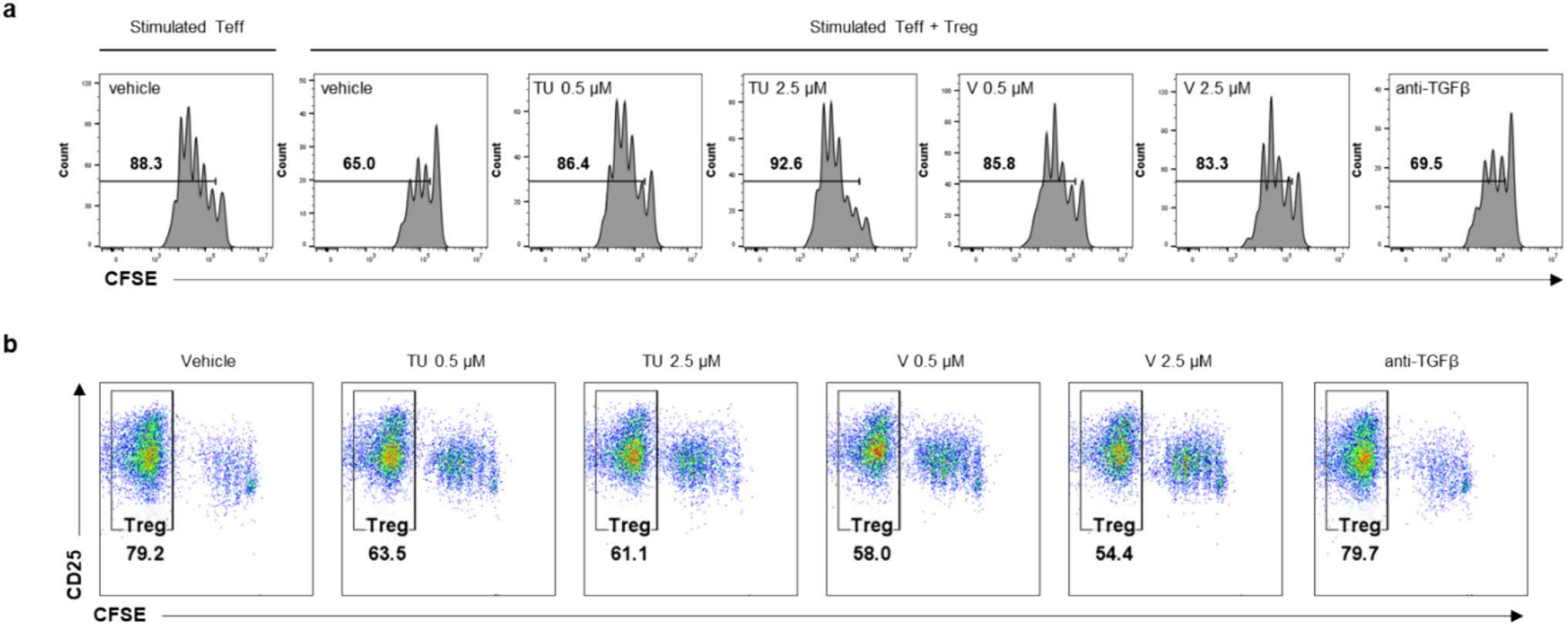
TU2218 blocks suppressive activity of regulatory T cell for proliferation of TCR-elicited effector T lymphocytes. **a** Activity of Treg suppression on proliferation of T effector cell (Teff). Histograms and % proliferation showing division of CFSE-labeled CD4^+^CD25^−^ Teff purified from pooled eight donor-derived PBMCs, cultured with anti-CD3 and anti-CD28 at Treg/Teff ratios of 3:1 for 5 days. Purified Teff cells without Treg cells (0:1) were used as a positive control of Teff proliferation. **b** Percentage of Treg (CD25 + CFSE-) after Treg and Teff coculture for 5 days. Noted TU indicates TU2218 and V indicates vactosertib and anti-TGFβ indicates anti-TGFβ neutralizing antibody

TGFβ is secreted from CD4 + CD25 + Tregs and is involved in maintaining Foxp3 expression in Tregs in an autocrine- or paracrine-dependent manner, and its biological role is a critical for mediating Treg-based immunosuppressive action [51]. We analyzed the effects of TU2218 treatment on Treg activity using a Treg suppression assay. In the Treg suppression assay, the proliferation of effector T cells (CD4 + CD25-), which was reduced by coculture with Tregs, was restored in a TU2218 concentration-dependent manner. This effect was also confirmed with vactosertib, but the efficacy of TGFβ neutralizing antibody was marginal (Fig. 3a). Interestingly, TU2218 seems to regulate not only Treg function but also Treg viability (Fig. 3b), and a therapeutic strategy based on small molecules that directly inhibits the activity of ALK5 is expected to be more effective at overcoming the immunosuppressive environment of Tregs than biologic drugs that antagonize TGFβ.

**Fig. 4:**
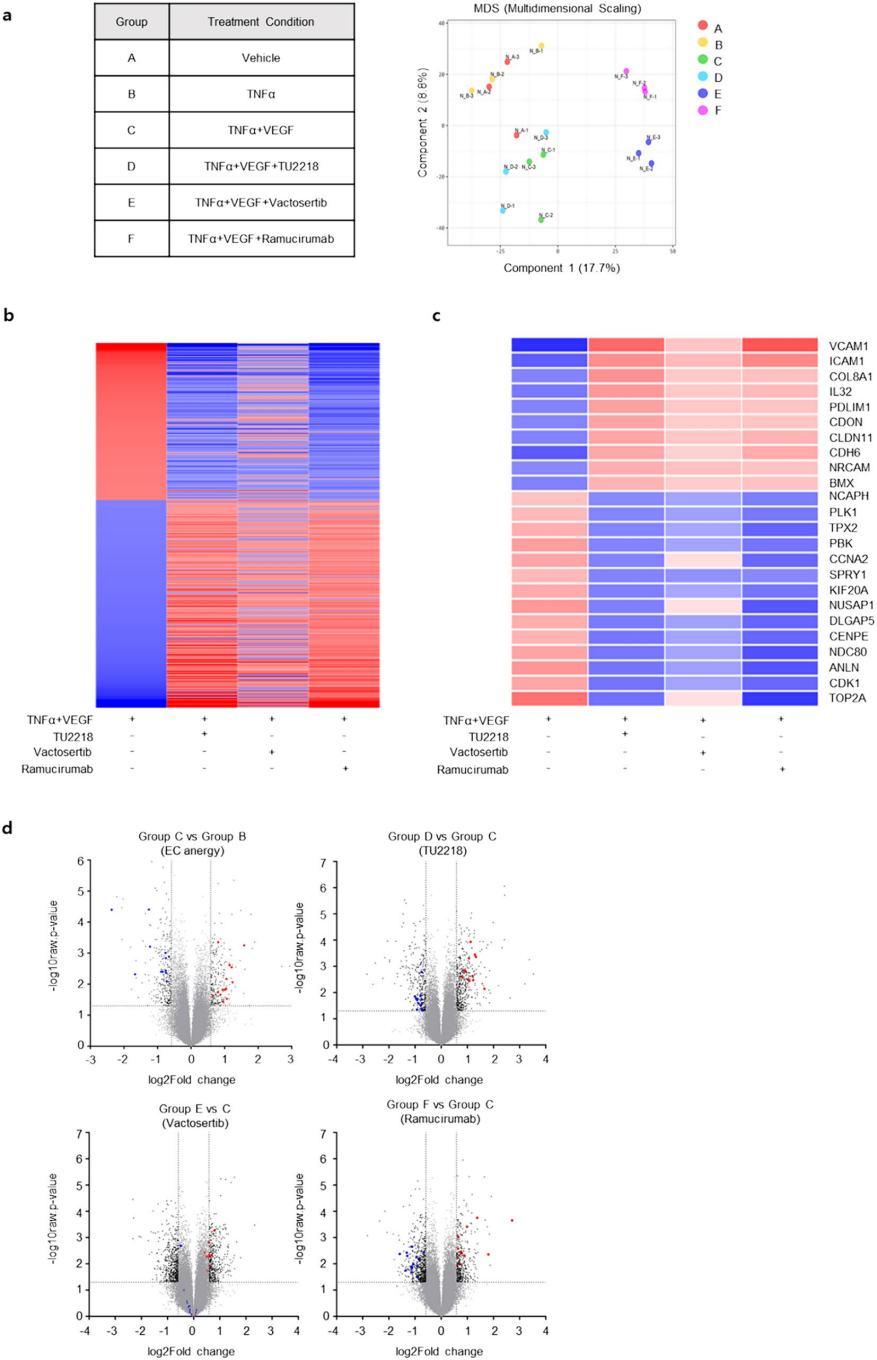

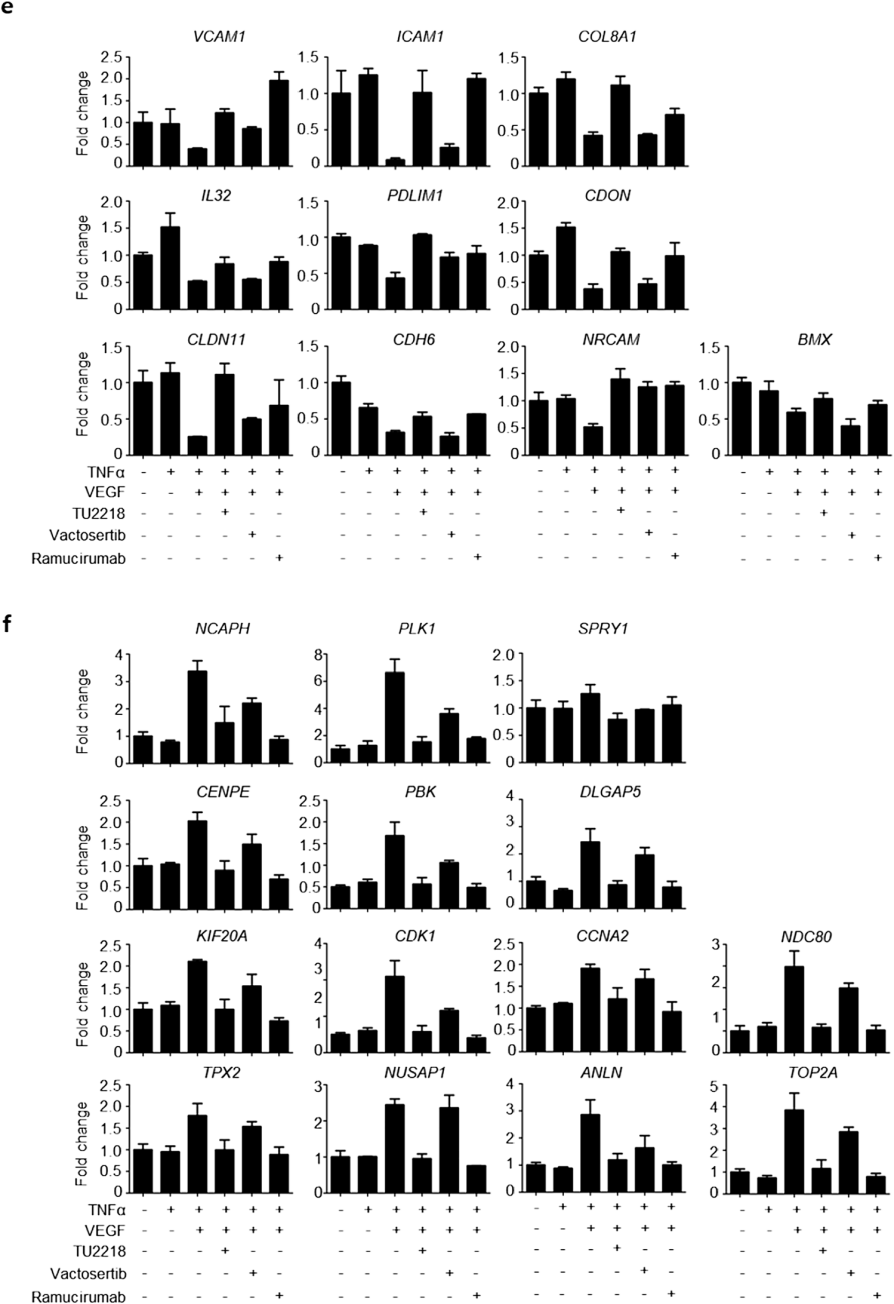

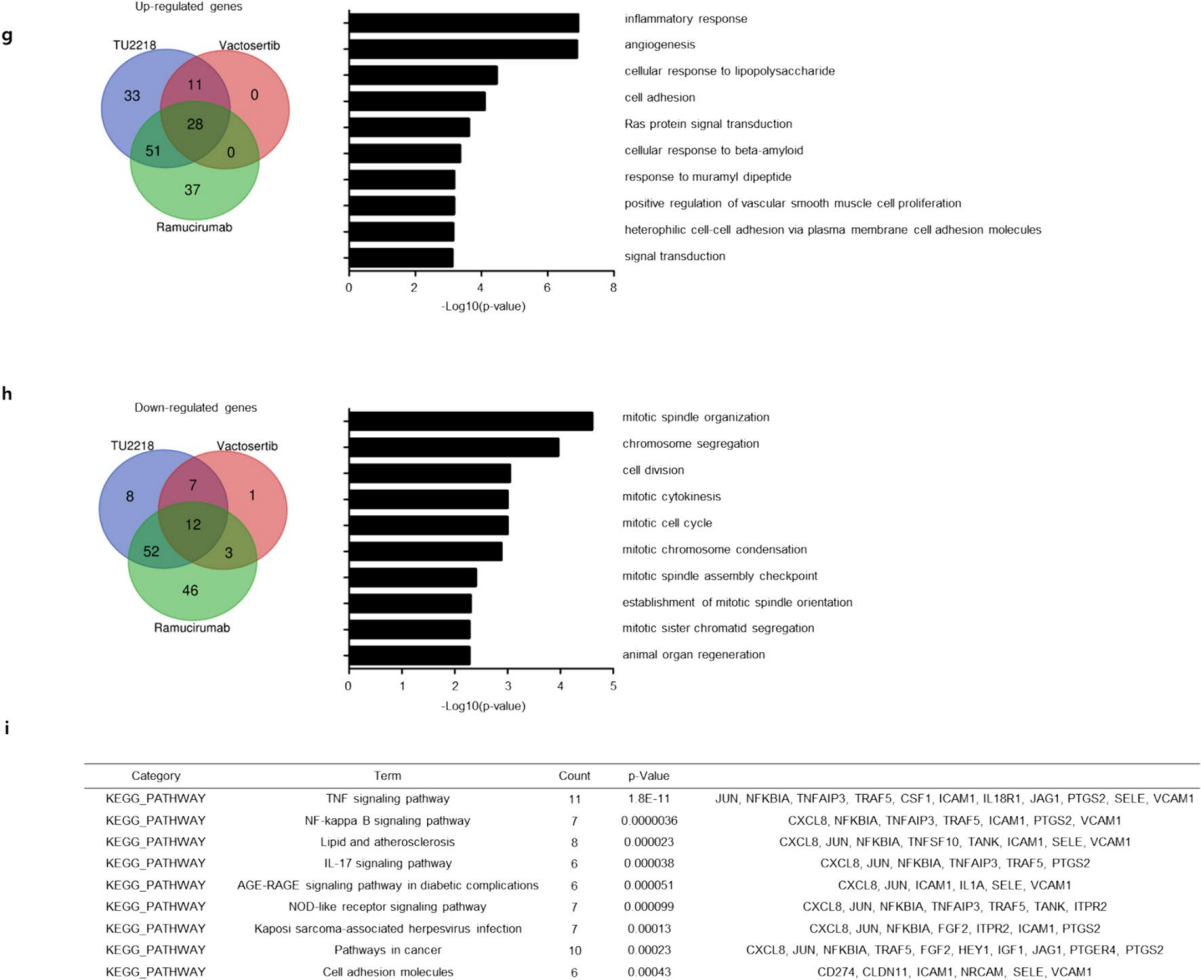
TU2218 normalizes the endothelial cell inactivation through inhibiting VEGF-VEGFR2 axis. **a** Overview of drug treatment condition (left) and multidimensional scaling (MDS) plot (right). **b** Heat map of 435 DEGs (fold change > 1.5 or < -1.5 and p ≤ 0.05) in TNFα + VEGF compared to those in TNFα. **c** Heat map of 24 DEGs related to cell adhesion or cell division. **d** Volcano plots of relative gene expression for indicated group comparison. 24 up- or downregulated genes are indicated in red or blue, respectively, showing distribution of 24 DEGs. **e**–**f** Relative mRNA expression of 24 DEGs confirmed by RT-PCR. **g**–**h** Dataset by Venn diagram of up- or downregulated genes by indicated treatment group (left). Enriched gene ontologies (GO, Biological Process) for overlap genes between TU2218 and ramucirumab. Lengths of the bars are indicative of* p* values for enrichment. Total list of gene ontology is in Supplementary Table S1 and S2 (right). (i) KEGG pathway analysis of overlap genes between TU2218 and ramucirumab using DAVID

TU2218 is a dual inhibitor that simultaneously inhibits ALK5 and VEGFR2 activity, and VEGFR2 inhibition by TU2218 is increased lymphocyte infiltration and improved endothelial cell inactivation. Recently, combination therapy with antiangiogenic agents and anti-PD-(L)1 blockade was confirmed to have breakthrough efficacy in various advanced carcinomas. The regulation of vascular-immune cross talk as a result of VEGFR activity suppression may be responsible for the successful combination treatment clinical trials, which is consistent with the effect of TU2218 on improving endothelial cell inactivation [27, 31–33, 35–37]. We experimentally mimicked the endothelial cell inactivation environment using human primary endothelial cells, HUVECs, and differentially expressed genes (DEGs) from TU2218 (an ALK5/VEGFR2 dual inhibitor), vactosertib (an ALK5 inhibitor), and ramucirumab (an anti-VEGFR2 antibody) treatments by microarray. As a result of the verified changes in 31,156 genes determined using 44,629 probes, 435 genes were identified as DEGs in TNFα and VEGF combination treatment compared to those in TNFα treatment. (Fig. 4a, b). DEGs were defined as those with a statistically significant 1.5-fold decrease or increase in gene expression. TU2218 treatment upregulated the expression of 123 genes and downregulated the expression of 79 genes against TNFα and VEGF combination treatment. The 123 upregulated genes included 10 common genes involved in cell adhesion. In addition, the 79 downregulated genes included 14 common genes involved in cell division (Fig. 4c). A comparative analysis of the distribution of 24 DEGs associated with cell adhesion and cell division functions using a volcano plot confirmed that the TU2218 and ramucirumab treatment groups had similar DEGs distribution, while that of the vactosertib treatment group was distinctly different from the other two groups (Fig. 4d). The difference in DEG distribution was explained by the degree to which each drug inhibited VEGFR2 activity. Among the analyzed DEGs, 10 with upregulated expression (*VCAM-1, ICAM-1, COL8A1, IL-32, PDLIM1, CDON, CLDN11, CDH6, NRCAM,* and *BMX*) and 14 with downregulated expression (*NCAPH, PLK1, SPRY1, CENPE, PBK, DLGAP5, KIF20A, CDK1, CCNA2, NDC80, TPX2, NUSAP1, ANLN*, and *TOP2A*) were verified using reverse transcriptase–polymerase chain reaction (Fig. 4e, f). A Venn diagram of the DEGs identified in the TU2218, vactosertib, and ramucirumab groups was constructed, and the results showed that the TU2218 group, which shared DEGs with the ramucirumab group, had a higher number of both upregulated and downregulated genes than the vactosertib group. In addition, when conducting a GO enrichment analysis on the shared genes between the TU2218 and ramucirumab groups, cell adhesion and cell division were found to be significantly enriched terms in the biological process category (Fig. 4g, h, Supplementary Tables S1, S2). In particular, according to the KEGG pathway analysis, *ICAM-1* and *VCAM-1* were confirmed to be significantly involved in cell adhesion (Fig. 4i).

**Fig. 5:**
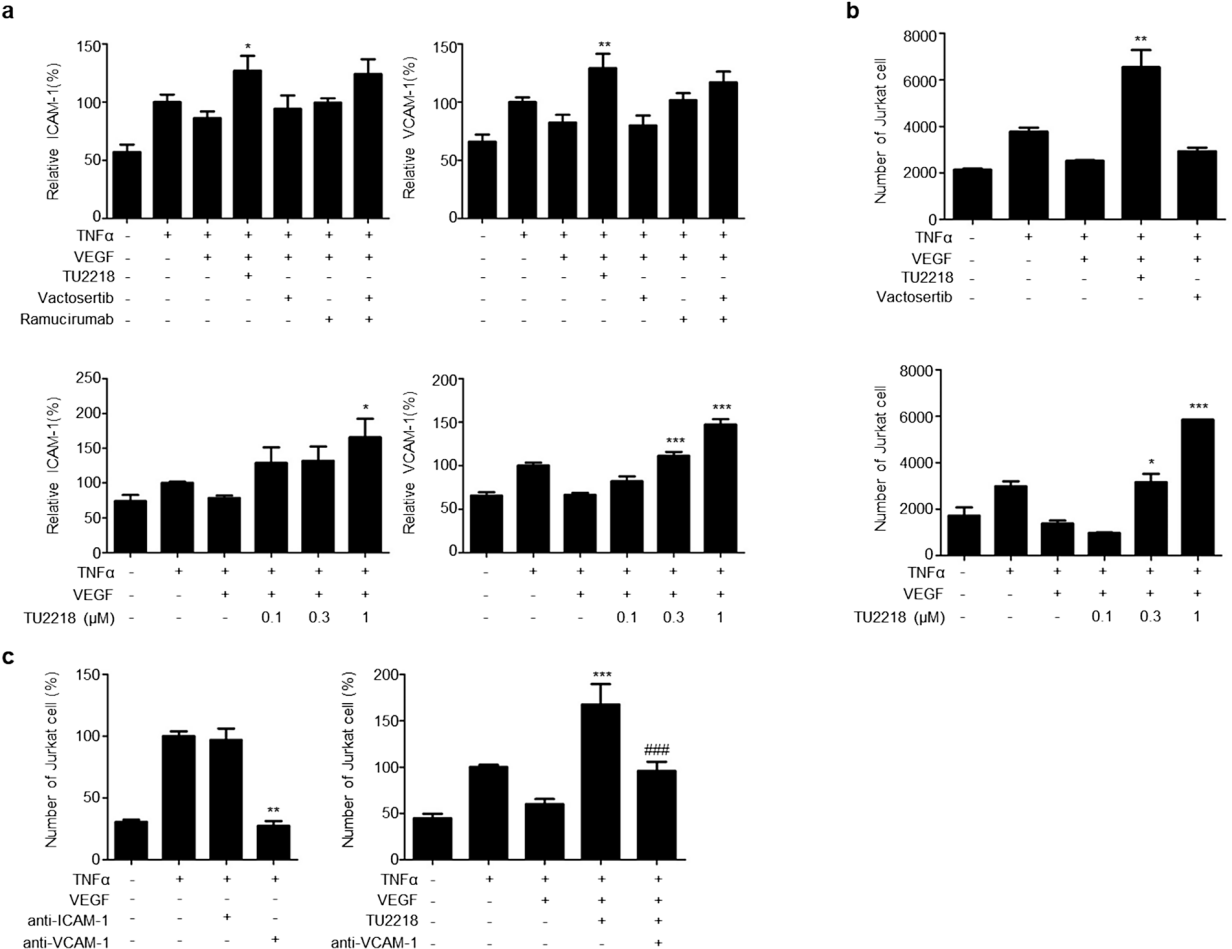
TU2218 improves Jurkat T adhesion on HUVECs monolayer in VEGF-induced endothelial inactivation. **a** Relative ICAM-1(%) and VCAM-1(%) expression by indicated treatment condition (upper) or dose-dependent TU2218 (bottom) compared to TNFα group. Mean fluorescence intensity (MFI) of surface ICAM-1 and VCAM-1 were assessed by flow cytometric analysis. **b**–**c** The number of adherent Jurkat T cells with indicated treatment. The number of Jurkat T cells was determined to count the CFSE-labeled Jurkat T cells bound on pre-treated HUVECs monolayer with indicated condition after coculture and removing unbound Jurkat T cells by flow cytometry. Blockade of ICAM-1 or VCAM-1 with neutralizing antibody (noted as anti-ICAM-1 or anti-VCAM-1) was used to prove biological function by TU2218 treatment. *p ≤ 0.05, **p ≤ 0.01, ***p ≤ 0.001 vs. TNFα + VEGF, ^###^*p* ≤ 0.001 vs. TNFα + VEGF + TU2218 (One-way ANOVA, Tukey)

The increase in the expression levels of these cell adhesion-related genes following TU2218 treatment and their involvement in improving endothelial cell inactivation was verified at the cellular level. In an experiment using HUVECs, ICAM-1 and VCAM-1 expression was downregulated after costimulation with TNFα and VEGF but significantly upregulated after TU2218 treatment. In contrast, ICAM-1 and VCAM-1 expression levels were not increased by vactosertib (Fig. 5a). To analyze the effect of TU2218 on cell adhesion, HUVECs were incubated with TNFα and VEGF and then cocultured with Jurkat T cells. The interaction between Jurkat T cells and HUVECs increased in response to TNFα stimulation alone but decreased in response to TNFα and VEGF costimulation. TU2218 treatment restored the reduced interaction between Jurkat T cells and HUVEC monolayers in a concentration-dependent manner, and this improvement in cell adhesion was mediated by VCAM-1, as confirmed by the use of a VCAM-1 neutralizing antibody (Fig. 5b, c, Supplementary figure S2a–e).

**Fig. 6:**
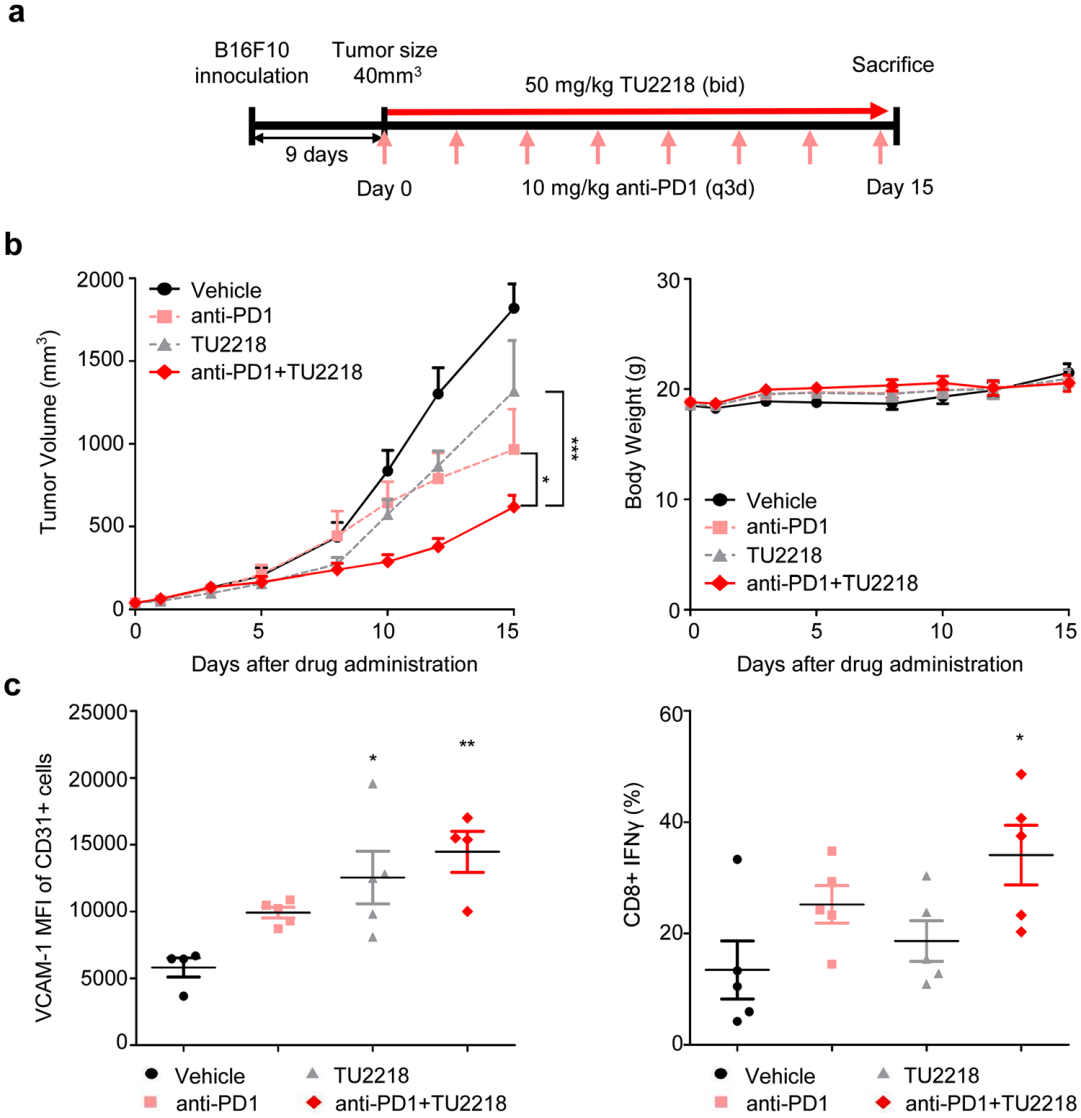
TU2218 induces antitumor activity in combination with anti-PD1 in B16F10 syngeneic model. **a** Experimental design of B16F10-bearing C57BL/6 mouse model. **b** Tumor volume at indicated time points. ***p ≤ 0.001 vs. TU2218, *p ≤ 0.05 vs. anti-PD1 (Two-way ANOVA) (left). Mean body weight at indicated time points (right). (c) MFI of VCAM-1 in CD31^+^ populations in tumor tissue. *p ≤ 0.05, **p ≤ 0.01 vs. vehicle (One-way ANOVA, Tukey) (left). Percent of CD8^+^IFNγ^+^ T cells in tumors. *p ≤ 0.05 vs. vehicle (One-way ANOVA, Tukey) (right). Data are shown as mean ± SEM

The in vivo antitumor efficacy of TU2218 was evaluated using a B16F10 syngeneic tumor model, which is a “cold” tumor type in which immune cells are deficient. Therefore, anti-PD1 therapy administered alone is unlikely to lead to significant therapeutic effects [52]. Considering these tumor characteristics, the B16F10 model was appropriate for confirming the synergistic effects of TU2218 and anti-PD1 on tumor-infiltrating lymphocyte (TIL) induction. Compared to the administration of anti-PD1 or TU2218 alone, a statistically significant reduction in tumor volume was observed after administration of the both anti-PD1 and TU2218. In addition, weight loss or toxicity due to drug administration was not observed during the drug administration (Fig. 6a, b). According to the flow cytometry analysis of tumor tissue, a significant increase in CD31 + VCAM-1 + cells was observed in the TU2218-administered group, and a significant increase in CD8 + T cells was observed in the anti-PD1 and TU2218-administered group (Fig. 6c).

**Fig. 7:**
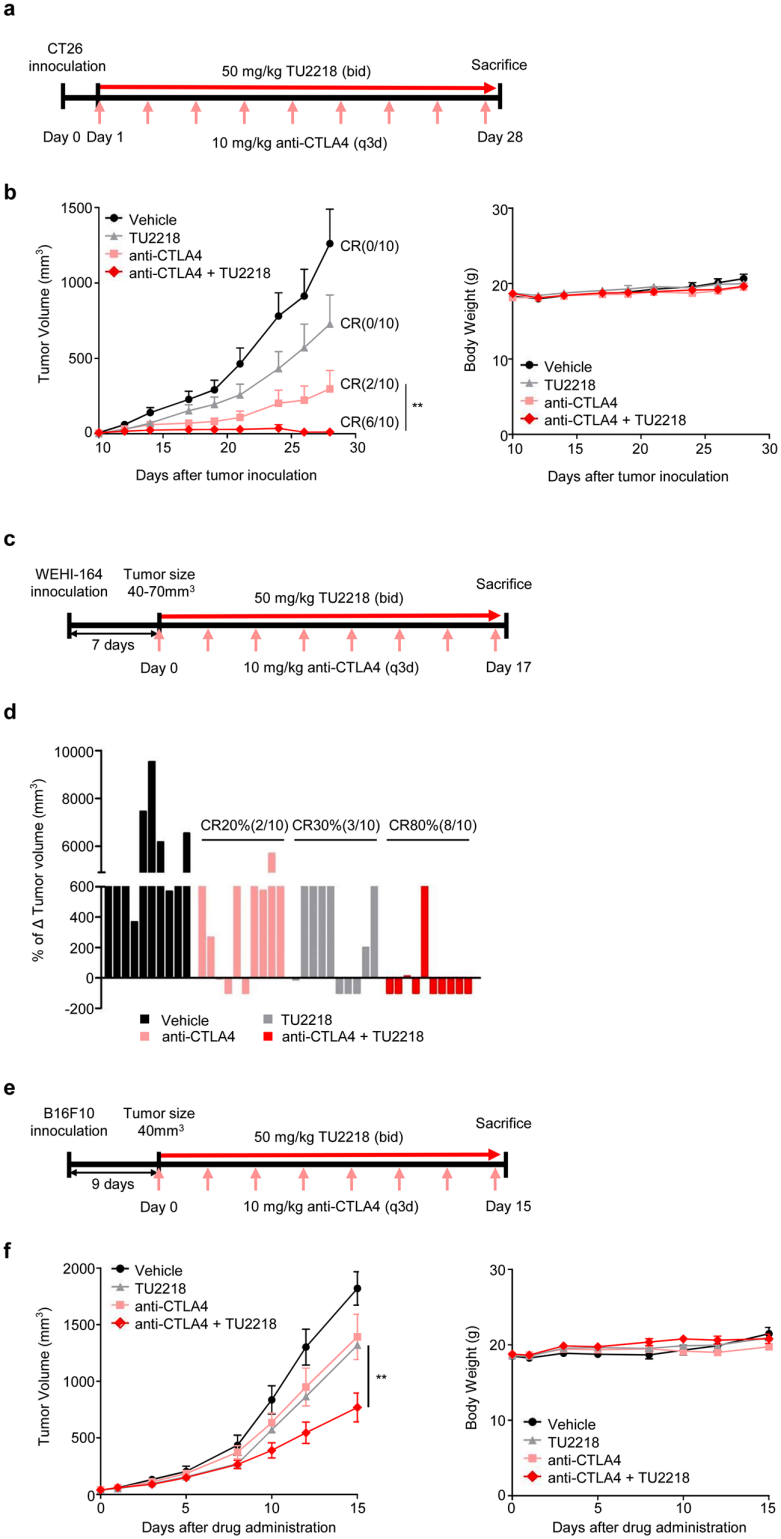

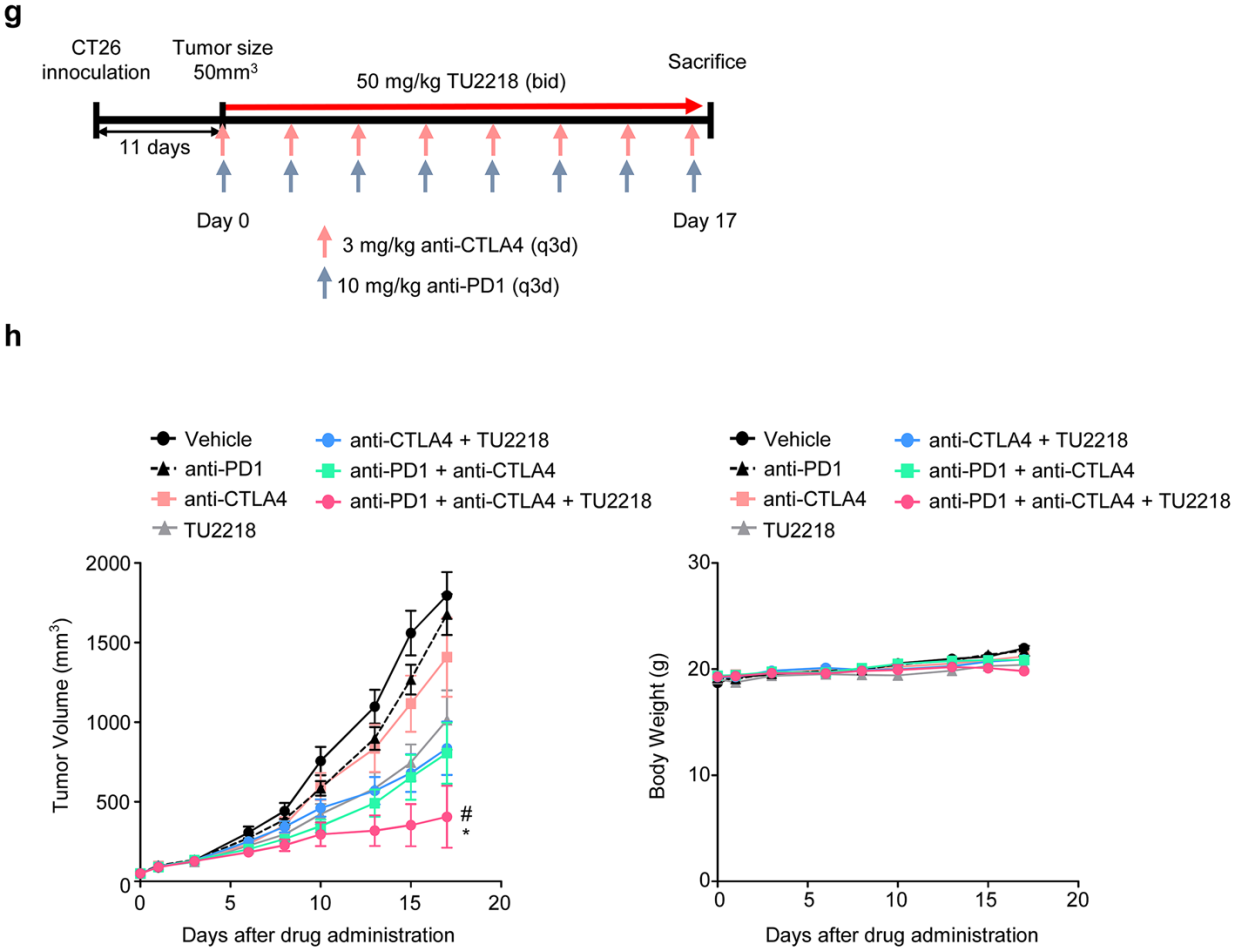
TU2218 shows synergistic antitumor effect with anti-CTLA4 in syngeneic tumor models. **a** Experimental design of CT26-bearing BALB/c mouse model. **b** Tumor volume or body weight at indicated time points; **p ≤ 0.01 vs. anti-CTLA4 antibody (Two-way ANOVA). **c** Experimental design of WEHI-164-bearing BALB/c mouse model. **d** % of Δ Tumor volume (mm^3^) at the endpoint (Day 17) normalized by tumor volume (mm^3^) at Day 0. And the CR (%) at the endpoint. **e** Experimental design of B16F10-bearing C57BL/6 mouse model. **f** Tumor volume and body weight at indicated time points; **p ≤ 0.01 vs. anti-CTLA4 antibody or TU2218 alone (Two-way ANOVA). **g** Experimental design of CT26-bearing BALB/c mouse model. **h** Tumor volume and body weight at indicated time points. ^#^*p* ≤ 0.05 vs. anti-PD1 + anti-CTLA4 antibody, and *p ≤ 0.05 vs. anti-CTLA4 antibody + TU2218 at Day 17 (Two-way ANOVA, Bonferroni post hoc test) (left). Data are shown as mean ± SEM

TU2218 may be able to exert antitumor effects in combination with various other drugs. In particular, the clinical application of the combination of the anti-CTLA4 antibody and TU2218 is prioritized among the list of possible combination regimens. In the CT26 and WEHI-164 syngeneic models, high frequency of complete regression (CR) of tumor was observed in anti-CTLA4 and TU2218 combination group, showing 60% (6/10), and 80% (8/10) of CR, respectively (Fig. 7a–d). Especially in the CT26 model, anti-CTLA4 and TU2218 combination showed significant antitumor effect compared to anti-CTLA4 or TU2218 monotherapy (Fig. 7b), but not in WEHI-164 model (data not shown). In the B16F10 syngeneic models, a significant antitumor effect was confirmed in the combination administration group compared with the anti-CTLA4 or TU2218 monotherapy groups (Fig. 7e, f). In addition, a triple combination of anti-PD1 antibody, anti-CTLA4 antibody, and TU2218 was analyzed in the CT26 syngeneic model, which showed a significantly higher antitumor effect than the anti-PD1 and anti-CTLA4 combination (Fig. 7g, h).

**Fig. 8:**
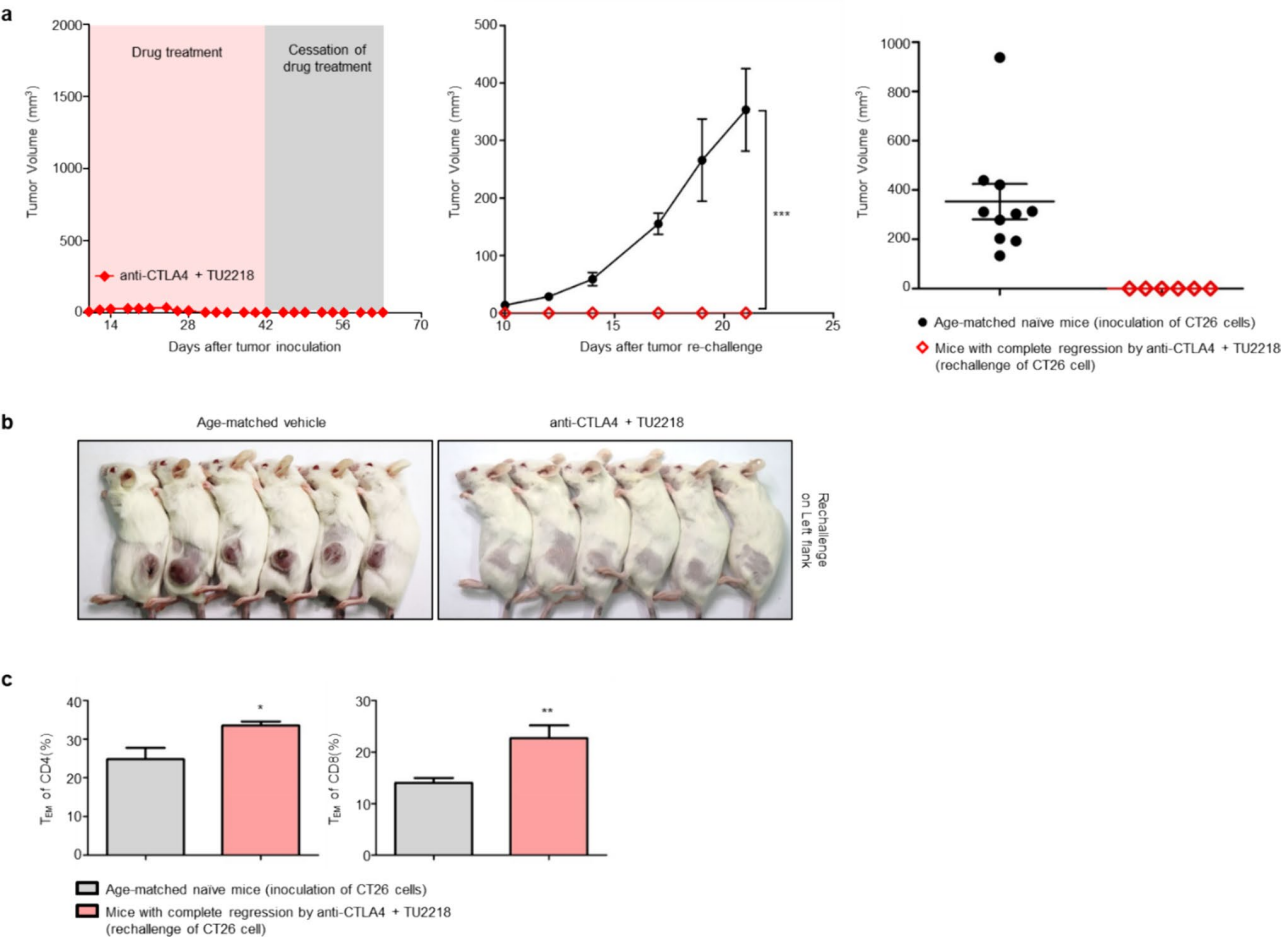
The combination with TU2218 and anti-CTLA4 leads to the formation of immunological memory. **a** Tumor volume during drug treatment and additional cessation of drug treatment (left). Tumor volume after CT26-rechallenge in tumor-eradicated mice showing CR by anti-CTLA4 + TU2218 administration and age-matched naïve mice at indicated timepoint (middle) or endpoint (right). After tumor eradication or CT26-rechallenge, mice were observed without drug administration to evaluate the formation of antitumor immunological memory. Data are shown as mean ± SEM. ***p ≤ 0.001 vs. age-matched naïve mice group (Two-way ANOVA). **b** Representative images of mice with CT26-rechallenge at endpoint. **c** The relative population of CD4^+^ and CD8^+^ T effector memory subsets in spleen of mice with CT26-rechallenge at endpoint. The data were obtained in six mice per group *p ≤ 0.05, **p ≤ 0.01 (*t*-test, Two-tailed)

Immunotherapy using immune checkpoint inhibitors is advantageous in terms of overall survival compared with chemotherapy or targeted therapy because of the formation of immunological memory, which prolongs clinical treatment efficacy [53, 54]. In the CT26 syngeneic tumor model, in which anti-CTLA4 and TU2218 were coadministered, complete regression was common, and a rechallenge assay was conducted to analyze whether immunological memory had been formed in these mice. After confirming CR by discontinuing drug administration, CT26 cells were transplanted into age-matched and CR-confirmed groups without additional drug administration (Fig. 8a, b). While tumors developed in every age-matched mouse, tumor formation was perfectly suppressed in the CR-confirmed individuals. In addition, the percentage of CD4 + and CD8 + effector memory T cells in the spleen was greater in the CR-confirmed group than the age-matched group (Fig. 8c, Supplementary figure S3a–c). Overall, these results demonstrate that TU2218-based combination strategies promote the formation of immunological memory.

## Discussion

The development of small-molecule compounds, antibodies, and fusion proteins that can target TGFβ has been ongoing for decades [55–57]. In the early stage of development of TGFβ inhibitors as anticancer drugs, the focus was on targeted anticancer agent for suppressing the growth of cancer cells through the regulation of TGFβ signaling; however, such a possibility has been demonstrated at a nonclinical level only [1, 2]. However, a deeper understanding of the tumor microenvironment owing to the development of immunotherapy led to the identification of TGFβ, which plays an important role in enabling cancer cells to escape the immune system by activating and differentiating the surrounding immune cells [13, 14, 44]. Furthermore, in a retrospective analysis of patients treated with anti-PD-(L)1 therapy, high levels of TGFβ were detected in the blood of patients who did not respond to anti-PD-(L)1 antibodies [8]. Additionally, inhibition of TGFβ signaling in nonclinical experiments has shown an increase in the efficacy of anti-PD-(L)1 antibodies [7]. These findings explain why TGFβ blockers should be developed as immune-related anticancer agents.

Analyzing the effect of TGFβ inhibitors on immunity is essential for understanding the mechanism of anticancer action, determining the effective concentration of drugs in the tumor microenvironment, and identifying unexpected off-target variables. Galunisertib is a representative small-molecule inhibitor of ALK5, and its effect has been analyzed in various immune cells [55]. Galunisertib treatment can improve TGFβ-mediated inhibition of CD8 + T lymphocyte proliferation and IFNγ expression and Treg-mediated inhibition of naïve T lymphocyte proliferation [58]. Galunisertib can also upregulate the expression of GzmB and IFNγ suppressed by TGFβ in NK cells [59]. The confirmed effective concentration that affects immune cells can be used to determine the appropriate dose during clinical development and for interpreting clinical efficacy. In contrast to vactosertib, galunisertib, and TGFβ neutralizing antibody drugs, TU2218 showed increased efficacy in improving endothelial cell inactivation and CTL activity. In addition, pharmacokinetic analysis proved that the current clinical administration dose of TU2218 can reach to the sufficient effective concentration for increasing CTL and NK activity and suppressing Tregs [42].

Anti-PD-(L)1 therapy has been shown to be effective in treating various cancer types. However, as monotherapy has a weak response rate, the need for combination therapy has emerged. The development of combination chemotherapy and targeted antiangiogenic drugs has significantly progressed [35–37, 60, 61]. In addition, an approach for discovering and resolving the cause of the low response to anti-PD-(L)1 therapy has been developed [62, 63]. The reduced IFNγ secretion observed in the coculture of PD-L1-expressing cancer cells and TCR-stimulated PBMCs was expected to be increased by pembrolizumab or atezolizumab treatment [49]. However, in our system, IFNγ secretion was not improved by the two immune checkpoint inhibitors, as shown in Fig. 2e. Interestingly, a clear recovery was observed with TU2218, and in particular, a tendency to improve IFNγ secretion by TGFβ neutralizing antibody was observed despite the absence of additional TGFβ stimulation. These findings suggest that TGFβ secreted in the presence of both cancer and immune cells can contribute to resistance to pembrolizumab and atezolizumab and may be related to the high expression of TGFβ in patients who do not respond to anti-PD-(L)1 treatment [7, 8]. Therefore, TGFβ signaling blockade by ALK5 inhibitors can be used as a breakthrough treatment for refractory and resistant patients to immune checkpoint inhibitors.

Tumors of the immune desert type are known to have low reactivity to anti-PD-(L)1 antibody drugs compared to inflamed type tumors. Major reasons for poor reactivity include low antigen presentation ability of tumor cells, limitation of penetration of immune cells due to activated stromal cells, reduction of intravascular adhesion proteins around tumors, and effect by chemokine [64]. The B16F10 syngeneic model is a representative immune desert model and was used to demonstrate the combination efficacy of TU2218 and anti-PD1 in Fig. 6. The background of statistically significant tumor reduction in the group administered with TU2218 and anti-PD1 antibodies compared to the anti-PD1 antibody administration group can be explained by positive correlation between an increase of adhesion molecules in vascular endothelial cells in tumors and infiltrated cytotoxic T lymphocyte in tumors. In addition to, role of chemokines has been reported to be involved in the process of recruitment of T lymphocyte into tumors. In particular, it has been reported that inhibition of TGFβ signaling increases CXCL9/10 in cancer-associated fibroblast subset, enhancing the infiltration of antigenic CD8 T lymphocyte in tumors [65], and CCL5 is known to induce apoptosis of CD8 T lymphocyte due to regulatory T lymphocyte by utilizing TGFβ as an effector [66]. Overall, the elevation of infiltrated T lymphocyte in tumors by TU2218 can be interpreted as a result of controlling both the adhesion molecule of endothelial cells and the chemokines in the tumor microenvironment. Incidentally, it is well known that the B16F10 syngeneic model hardly reacts with anti-PD1 alone, but the background of partial response derived with anti-PD1 alone in Fig. 6 seems to be related to starting drug administration at a palpable tumor size of less than 50 mm^3^. Several studies using the B16F10 syngeneic model also confirmed the partial efficiency of anti-PD1 alone to a similar level [67–69].

Our results provide evidence to explain the synergistic compatibility and clinical effects of the combination of TU2218 and anti-PD-(L)1 agent. Two representative functions of TU2218 that support synergy with anti-PD-(L)1 therapy are the inhibition of Treg activity, which is governed by TGFβ-ALK5, and the induction of TILs through improvements in endothelial cells, which are governed by VEGF-VEGFR2. In fact, a multityrosine kinase inhibitor that inhibits VEGFR2, or ipilimumab, which inhibits Treg activity, combined with anti-PD1 therapy has shown clinical benefits in several cancers [35]. This finding demonstrates that overcoming immune evasion and immune tolerance caused by VEGFR2 and TGFβ in the tumor microenvironment is essential for ensuring clinical efficacy. In addition, we are interested in a clinical study on the combination of ipilimumab and pembrolizumab in patients with melanoma that are unresponsive to anti-PD-(L)1 therapy [70]. Our focus in this clinical trial was that a meaningful response of 7.2% CR and 21.4% partial regression was induced in PD-(L)1 therapy-resistant patients. Moreover, the anti-CTLA4 antibody-based combination strategy could be a breakthrough for patients who are resistant to anti-PD-(L)1 therapy [70]. In syngeneic tumor models, we observed that the combination of TU2218 and an anti-CTLA4 antibody not only induced tumor CR but also completely eradicated repeated tumor challenges through the formation of immunological memory. This combination option is valuable and may be developed as a therapeutic alternative in the future for patients unresponsive to anti-PD-(L)1 therapy.

Overall, TU2218 is a dual inhibitor that simultaneously inhibits ALK5 and VEGFR2 and is expected to create a favorable environment for immune checkpoint inhibitors to act by controlling various immunosuppressive factors in the tumor microenvironment. This mechanism of action of TU2218 may overcome the limitations of immune checkpoint inhibitor monotherapy.

## Materials and methods

### Reagent

The ALK5 inhibitor TU2218, vactosertib, or galunisertib was obtained from TiumBio (Gyeonggi-do, Korea, TU2218 was developed by TiumBio, and phase 1a, the monotherapy of TU2218, has been terminated and phase 1b, a combination study with pembrolizumab, is nearing the end. A combination study of phase 2a with pembrolizumab is scheduled in the third quarter of this year (NCT05204862, NCT05784688)), BLD Pharmatech (Shanghai, China), or Crysdot (Bel Air, MD USA). Recombinant human TGFβ, VEGF, and anti-TGFβ neutralizing antibody (clone 1D11) were obtained from R&D systems (Minneapolis, MN, USA). Pembrolizumab, atezolizumab, and ramucirumab were obtained from Selleckchem (Houston, TX, USA). For in vivo administration, anti-mouse PD1 (clone RMP1-14) and anti-mouse CTLA4 antibodies (clone 9D9) were obtained from BioXCell (Lebanon, NH, USA). Anti-CD3/CD28 coated-microbead was prepared from T cell activation/expansion kit (Miltenyi Biotec, Bergisch Gladbach, Germany) according to the manufacturer’s protocol. Fetal bovine serum (FBS) was obtained from SERANA (Brandenburg, Germany). Phospho-buffered saline (PBS), Hanks' balanced salt solution (HBSS), and trypsin–EDTA were provided from Lonza (Basel, Switzerland) or Thermo Fisher Scientific (Massachusetts, USA).

### Cell culture

Human PBMCs were purchased from STEMCELL Technology (Vancouver, Canada). Cells were cultured with RPMI-1640 media (Welgene, Gyeongsangbuk-do, Korea) containing 10% heat-inactivated FBS. MCF-7, HT-1080, K562, and Jurkat T cells were obtained from Korean Cell Line Bank, KCLB (Seoul, Korea). NK92mi, CT26, and HUVEC were obtained from American Type Culture Collection, ATCC (Manassas, VA, USA). Cells were incubated at 37℃, 5% CO_2_ humidified incubator.

### Enzyme assay for ALK5 and VEGFR2

Each compound was tested against indicated kinases (Reaction Biology Corporation, Malvern, PA, USA). Compounds were treated to 10 points with a threefold serial dilution starting at 10 μM or 100 μM. Enzyme reactions were performed with 10 μM ATP and buffer (20 mM HEPES, pH 7.5, 10 mM MgCl_2_, (2 mM MnCl_2_ if applicable), 1 mM EGTA, 0.02% Brij 35, 0.02 mg/ml bovine serum albumin (BSA), 0.1 mM Na_3_VO_4_, 2 mM DTT, and 1% DMSO) for 2 h.

### Phospho-SMAD2/SMAD2 assay

Human whole blood was treated with various concentration of TU2218 and 5 ng/mL of TGFβ was stimulated for additional 30 min. Reaction was stopped with Lyse/Fix buffer (BD bioscience, NJ, USA) and cells were permeabilized by Perm III buffer (BDbioscience) and stained using the following antibodies: anti pSMAD2(Ser465/467)/SMAD3(pS423/pS425)-PE (clone O72-670, BD Bioscience) or anti-SMAD2-AF647 (clone D43B4, Cell Signaling, Danvers, MA, USA). Stained cells were analyzed with CytoFLEX flow cytometer (Beckman Coulter, Brea, CA, USA) and data were analyzed by Cytpert or Prism 5 software (GraphPad, Boston, MA, USA). The relative ratio of pSMAD2/SMAD2 was calculated using the following equation (pSMAD2 of vehicle = 0%, pSMAD2 of TGFβ = 100%).

$${\text{Relative}}\,{\text{ratio}}\,{\text{pSMAD}}2/{\text{SMAD}}2_{{\text{of}}\,{\text{indicated}}\,{\text{treatment}}\,{\text{condition}}}=\frac{{{\text{Average}}\,{\text{of}}\,{\text{pSMAD}}2\,{\text{MFI}}/{\text{SMAD}}2\,{\text{MFI}}_{{\text{of}}\,{\text{vehicle}}}-{\text{Average}}\,{\text{of}}\,{\text{pSMAD}}2\,{\text{MFI}}/{\text{SMAD}}2\,{\text{MFI}}_{{\text{of}}\,{\text{indicated}}\,{\text{treatment}}\,{\text{condition}}}}}{{{\text{Average}}\,{\text{of}}\,{\text{pSMAD}}2\,{\text{MFI}}/{\text{SMAD}}\,2{\text{MFI}}_{{\text{of}}\,{\text{vehicle}}}}-{\text{Average}}\,{\text{of}}\,{\text{pSMAD}}2\,{\text{MFI}}/{\text{SMAD}}2\,{\text{MFI}}_{{\text{of}}\,{\text{TGF}}\beta }} \times 100$$

All human samples were prospectively obtained under written informed consent. All study protocols were approved by the Institutional Review Board of Tiumbio Co., Ltd (IRB code: TBIRB-22-BR-001).

### Phospho-VEGFR2/VEGFR2 assay

The HUVECs were seeded on gelatin (Sigma, St. Louis, MO, USA)-coated 12-well plates. Cells were starved for overnight and treated with various concentration of compound for 1 h and then stimulated with 20 ng/mL VEGF for 15 min. Cells were lysed in RIPA buffer (Sigma) containing protease inhibitors (cOmplete mini, EDTA-free; Roche, Basel, Switzerland) and phosphatase inhibitor cocktail (PhosStop, Roche). Lysates were analyzed to immunoblotting assay using anti-phospho VEGFR2 antibodies (clone 19A10, Cell Signaling) and anti-VEGFR2 (clone D5B1, dilution 1: 1000) (Cell Signaling). Semiquantitative densitometry was performed using Image J software (NIH, Bethesda, MD, USA). And IC_50_ was calculated to Prism 5 Software.

### TGFβ-induced immune suppression

PBMCs were seeded on 96-well plate and treated with various concentration of test drugs with 5 or 50 ng/mL TGFβ, and anti-CD3/CD28 microbead. To analyze intracellular IFNγ, PBMCs were incubated with adding Golgistop (BD bioscience) before collection of cells and stained with the following antibodies (surface): anti-human CD3-APC-Cy7 (clone UCHT1), anti-human CD8a-AF700 (clone RPA-T8), and anti-human CD4-PE-Cy7 (clone RPA-T4), anti-human IFNγ-PE-CF594 (clone B27, Biolegend). To measure secreted-IFNγ, supernatants were analyzed by IFNγ ELISA kit (Thermofisher Scientific). To detect compound effect on natural killer (NK) cell in PBMC or NK92mi, cells were stimulated with 5 ng/mL TGFβ with various concentration of test drugs. Cells were stained by anti-human NKG2D-PE (clone 1D11) and anti-human CD56-PB (clone 5.1H11). Expression pattern of NK2GD was analyzed on CD56 bright or CD56 dim population. Data were analyzed by Cytpert or Flow Jo v10.7.2 (FlowJo, Ashland, OR, USA).

### Coculture

Human PBMCs were cocultured with MCF-7 or HT-1080 at 2:1 or 4:1 ratio respectively, and treated with various concentration of test drugs, and followed by adding anti-CD3/CD28 microbead. The supernatant was collected after 48 h and applied to IFNγ ELISA method (Invitrogen). Preincubated-NK92mi cells with TGFβ and test drug were cocultured with CFSE(Invitrogen)-labeled K562 cells at ratio 2:1 for 24 h and cytocidal activity of NK92mi cells was analyzed to quantification of CFSE-labeled K562 using flow cytometry.

### Treg suppression assay

Regulatory T cell (Treg) and effector T cell (Teff) were isolated from pooled PBMCs from 8 healthy donors using CD4 + CD25 + Regulatory T cell isolation kit (Miltenyi Biotec) according to manufacturer’s protocol. CFSE-labeled Teff was incubated on 96-well with Treg cells at 1:3 ratio, with various concentration of test drugs. All cells were stimulated with anti-CD3/CD28 microbead for 5 days. To measure Teff proliferation, cells were stained with anti-human CD25-PE (clone BC96, Biolegend) and CFSE dilution was analyzed using flow cytometry.

### Endothelial cell inactivation

HUVECs were starved in serum-free EBM-2 media for overnight and treated with various concentration of test drugs and then stimulated with 100 ng/mL VEGF and 0.04 ng/mL TNFα (Peprotech, New Jersey, US). To analyze the level of surface ICAM-1 and VCAM-1, cells were stained with anti-human ICAM-1-PE (clone HA58) and anti-human VCAM-1-APC (clone STA) (Biolegend). The expression level of ICAM-1 and VCAM-1 was measured to flow cytometry. The relative ICAM-1(%) or VCAM-1(%) was calculated using the following equation (TNFα = 100%).

$${\text{Relative}}\,{\text{ICAM}} - 1\left( {{\text{VCAM}} -1}\right){\left( {{\%}}\right)_{{\text{of}}\,{\text{indicated}}\,{\text{treatment}}}}=\frac{{{\text{Average}}\,{\text{of}}\,{\text{ICAM}} -1\left({{\text{VCAM}} -1}\right)\,{\text{MF}}{{\text{I}}_{{\text{of}}\,{\text{indicated}}\,{\text{treatment}}}}}}{{{\text{Average}}\,{\text{of}}\,{\text{ICAM}}-1\,{\text{or}}\,{\text{VCAM}}-1\,{\text{MF}}{{\text{I}}_{{\text{of}}\,{\text{TNF}}\alpha }}}}\times 100$$

To analyze binding activity of Jurkat on anergic HUVECs, the pre-treated HUVECs with VEGF and TNFα were cocultured with CFSE-labeled Jurkat cells at 1:1 ratio for 25 min. And then unbound Jurkat cells were washed and bound Jurkat cells were harvested to 0.05% trypsin–EDTA. CFSE-labeled Jurkat cells were isolated on HUVECs and Jurkat mixture to flow cytometry.

#### Microarray analysis

Microarray was conducted using GeneChip Human Gene 2.0ST Array platform (Macrogen Corporation, Seoul, Korea). RNA samples were obtained from triplicate repeated-experiments. Total RNA was extracted from HUVECs using Direct-zol RNA MiniPrep (Zymoresearch, Irvine, CA, USA) according to the manufacturer’s recommendations. Complementary DNA (cDNA) was synthesized using the GeneChip WT (Whole Transcript) Amplification kit as described by the manufacturer. The sense cDNA was then fragmented and biotin-labeled with TdT (terminal deoxynucleotidyl transferase) using the GeneChip WT Terminal labeling kit. Approximately 5.5 μg of labeled DNA target was hybridized to the Affymetrix GeneChip Array at 45 °C for 16 h. Hybridized arrays were washed and stained on a GeneChip Fluidics Station 450 and scanned on a GCS3000 Scanner (Affymetrix). The probe cell intensity data computation and a CEL file generation were performed using Affymetrix® GeneChip Command Console® Software (AGCC). Differentially expressed genes (DEGs) data were considered significant if both the p value ≤ 0.05, fold change (FC) < -1.5 or > 1.5 after normalization using AGCC. The gene ontology (GO) annotations of the DEGs were downloaded from the DAVID Bioinformatics Resources (https://david.ncifcrf.gov). The pathway analysis was obtained from the Kyoto Encyclopedia of Genes and Genomes (KEGG) database (http:// www.genome.jp/kegg). qRT-PCR was performed using iQ SYBR Green Supermix (BioRad, Hercules, CA, USA) following the instructions. Glyceraldehyde-3-phosphate dehydrogenase (GAPDH) was used as a housekeeping gene. Data were examined by the 2-ΔΔCt method. Primers for qRT-PCR are listed in the Supplementary Table S3.

#### Syngeneic mouse tumor models and tumor tissue analysis

C57BL6 and BALB/c female mice between 6 and 8 weeks were obtained OrientBio (Gyeonggi-do, Korea) or Vital River Laboratories Research Models and Services (Beijing, China). Each mouse was subcutaneously inoculated with B16F10(3 × 10^5^ cells), CT26(5 × 10^5^ cells), and WEHI-164(1 × 10^6^ cells) tumor cells in the flank. When tumor size reached approximately 30–60 mm^3^, mice were randomized and then drug treatments were continued for indicated periods. Tumor size was measured three times a week and calculated by the following formula: Volume = (Length × Width × Width)/2. Mice were housed under pathogen-free condition with a maximum of 5 per cage. For Fig. 7d, increase/decrease percentage analysis of tumor volume at day 17 was calculated by the following equation:

$$\% \,{\text{of}}\,{{\Delta }}\,{\text{Tumor}}\,{\text{volume}}\,\left({{\text{mm}}^3} \right) =\frac{{{\text{tumor}}\,{\text{volume}}{{\left({{\text{mm}}^3}\right)}_{{\text{at}}\,{\text{Day}}\,17}} -{\text{tumor}}\,{\text{volume}}\,{{\left({{\text{mm}}^3}\right)}_{{\text{at}}\,{\text{Day}}\,0}}}}{{{\text{tumor}}\,{\text{volume}}\,{{\left({{\text{mm}}^3}\right)}_{{\text{at}}\,{\text{Day}}\,0}}}} \times 100$$

Biopsied tumor tissues were dissociated with Tumor dissociation kit (Miltenyi Biotec) and gentleMACS (Miltenyi Biotec). Erythrocytes were lysed and single-cell suspensions were incubated with Fc block and stained with the following antibodies: anti-mouse CD31-FITC (clone 390, eBioscience), anti-mouse VCAM-1-APC (clone 429, Biolegend) anti-mouse CD45-BV510 (clone 30-F11, BD Bioscience), anti-mouse CD3-APC-Cy7 (clone 145-2C11, Invitrogen), anti-mouse CD8-BB515 (clone 53–6.7, BD Bioscience), and anti-mouse IFNγ-APC (clone XMG1.2, BD Bioscience). Samples were analyzed to flow cytometer and data were analyzed by Cytpert or FlowJo v10.7.2.

To detect immunological memory activity, both complete regressed-mice of TU2218 plus anti-CTLA4 mAb combination group and age-matched mice were rechallenged with CT26 (5 × 10^5^ cells) on the opposite flank. And then tumor generation and size were observed without drug treatment. For immune profiling of spleen tissue, spleens were harvested and mechanically dissociated to acquire single-cell suspensions using a plunger. Cells were stained with following antibodies: anti-mouse CD45-BV510 (clone 30-F11, BD Bioscience), anti-mouse CD3-APC-Cy7 (clone 145-2C11, Invitrogen), anti-mouse CD8-BB515 (clone 53–6.7, BD Bioscience), anti-mouse CD4-PE-Cy7 (clone RM4-5, BD Bioscience), anti-mouse CD44-PE (clone IM7, BD Bioscience), and anti-mouse CD62L-APC (clone MEL-14, Biolegend). Samples were analyzed to flow cytometer and data were analyzed by FlowJo v10.7.2. All protocols for animal models were approved by the Institutional Animal Care and Use Committee (IACUC) of Chaon Inc (Gyeonggi-do, Korea) or Crown Bioscience Inc. (Beijing, China).

#### Statistical and data analysis

All statistical analyses were processed using GraphPad Prism 5 (Boston, MA). Statistical analysis with multiple groups was completed using one-way ANOVA with post hoc Tukey test or two-way ANOVA with post hoc Bonferroni test. Data are represented as the mean with error bars signifying the standard error of the mean (SEM) unless otherwise indicated. Statistical significance is indicated as *p < 0.05, **p < 0.01, ***p < 0.001. Gating strategy and representative FCM graph were included in indicated Supplementary figures.

## Supplementary Information

Below is the link to the electronic supplementary material.

Supplementary file1 (PDF 1143 kb)

## Data Availability

The datasets generated during and/or analyzed during the current study are available from the corresponding author on reasonable request. The microarray data from this research are available at GEO accession number GSE239760 (https://www.ncbi.nlm.nih.gov/geo/query/acc.cgi?acc=GSE239760).

## Abbreviations

ANLN: Anillin, actin binding protein
b.i.d: Two times a day
BMX: BMX non-receptor tyrosine kinase
COL8A1: Collagen type VIII alpha 1 chain
CDON: Cell adhesion associated, oncogene regulated
CLDN11: Claudin-11
CENPE: Centromere protein E
CDK1: Cyclin-dependent kinase 1
CCNA2: Cyclin A2
CDH6: Cadherin-6
DLGAP5: DLG-associated protein 5
Foxp3: Forkhead box P3
GzmA: Granzyme A
GzmB: Granzyme B
ICAM-1: Intercellular adhesion molecule 1
IL-32: Interleukin 32
KIF20A: Kinesin family member 20A
NRCAM: Neuronal cell adhesion molecule
NCAPH: Non-SMC condensin I complex subunit H
NDC80: NDC80 kinetochore complex component
NUSAP1: Nucleolar and spindle-associated protein 1
PDLIM1: PDZ and LIM domain 1
PLK1: Polo-like kinase 1
PBK: PDZ binding kinase
SDS-PAGE: Sodium dodecyl sulfate–polyacrylamide gel electrophoresis
SPRY1: Sprouty RTK signaling antagonist 1
TPX2: TPX2 microtubule nucleation factor
TOP2A: DNA topoisomerase II alpha
TNFα: Tumor necrosis factor α
VCAM-1: Vascular cell adhesion molecule 1
qRT-PCR: Real-time quantitative reverse transcription PCR
