# Supplementary material for: ALK5/VEGFR2 dual inhibitor TU2218 alone or in combination with immune checkpoint inhibitors enhances immune-mediated antitumor effects
Source: Cancer Immunol Immunother. 2024 Aug 6;73(10):190. doi: 10.1007/s00262-024-03777-4 (PMC11303640; doi:10.1007/s00262-024-03777-4)
Supplement: Supplementary file 1 — Supplementary file1 (PDF 1143 kb) [file 262_2024_3777_MOESM1_ESM.pdf]

Supplementary Figure S1. Gating strategy and FCM graph for Figure 2

(a) Gating strategy of CD56dim or CD56bright NK cells in PBMC for Figure 2c

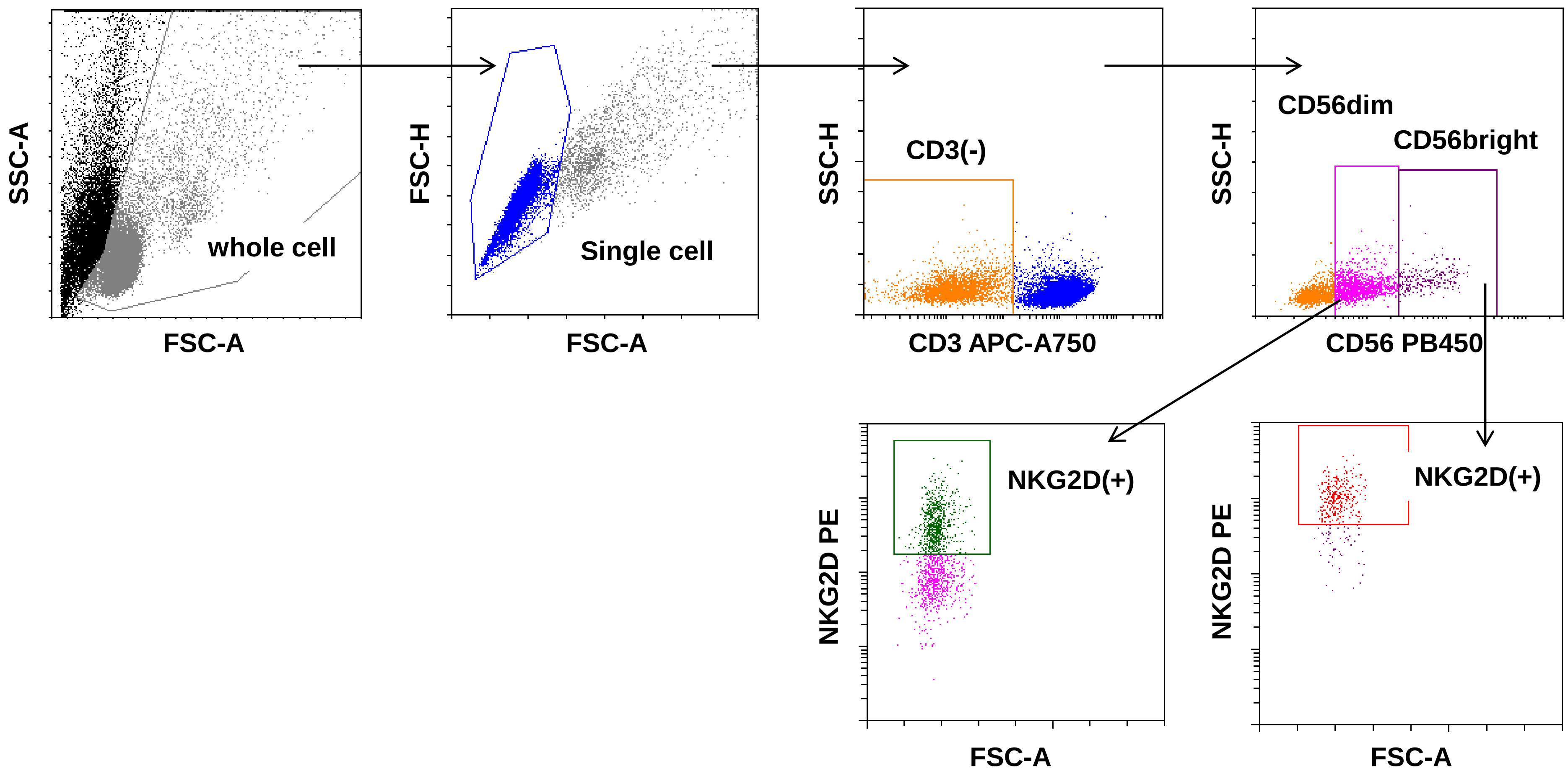

Supplementary Figure S1. Gating strategy and FCM graph for Figure 2

(b) Representative FCM graph for Figure 2c (left, CD56dim)

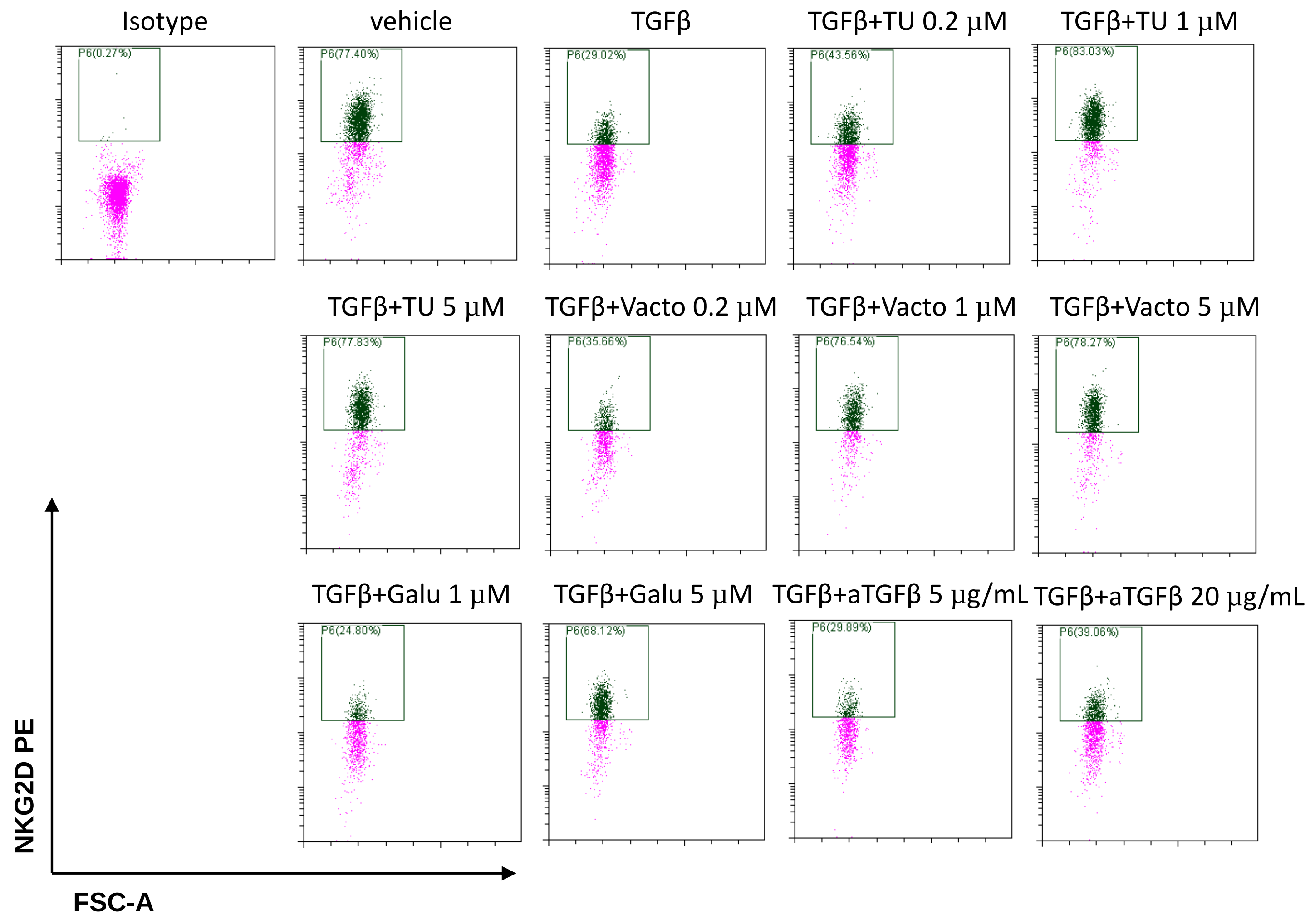

Group	Cell (%)	Relative Cell (%)
Isotype	0.27	-
vehicle	77.40	100.0
TGFβ	29.20	37.7
TGFβ+TU2218 0.2 μM	43.56	56.3
TGFβ+TU2218 1 μM	83.03	107.3
TGFβ+TU2218 5 μM	77.83	100.6
TGFβ+Vactosertib 0.2 μM	35.66	46.1
TGFβ+Vactosertib 1 μM	76.54	98.9
TGFβ+Vactosertib 5 μM	78.27	101.1
TGFβ+Galunisertib 1 μM	24.80	32.0
TGFβ+Galunisertib 5 μM	68.12	88.0
TGFβ+anti-TGFβ 5 μg/mL	29.89	38.6
TGFβ+anti-TGFβ 20 μg/mL	39.06	50.5

Abbreviation; TU; TU2218, Vacto; Vactosertib, Galu; Galunisertib, aTGFβ; anti-TGFβ

(c) Representative FCM graph for Figure 2c (right, CD56bright)

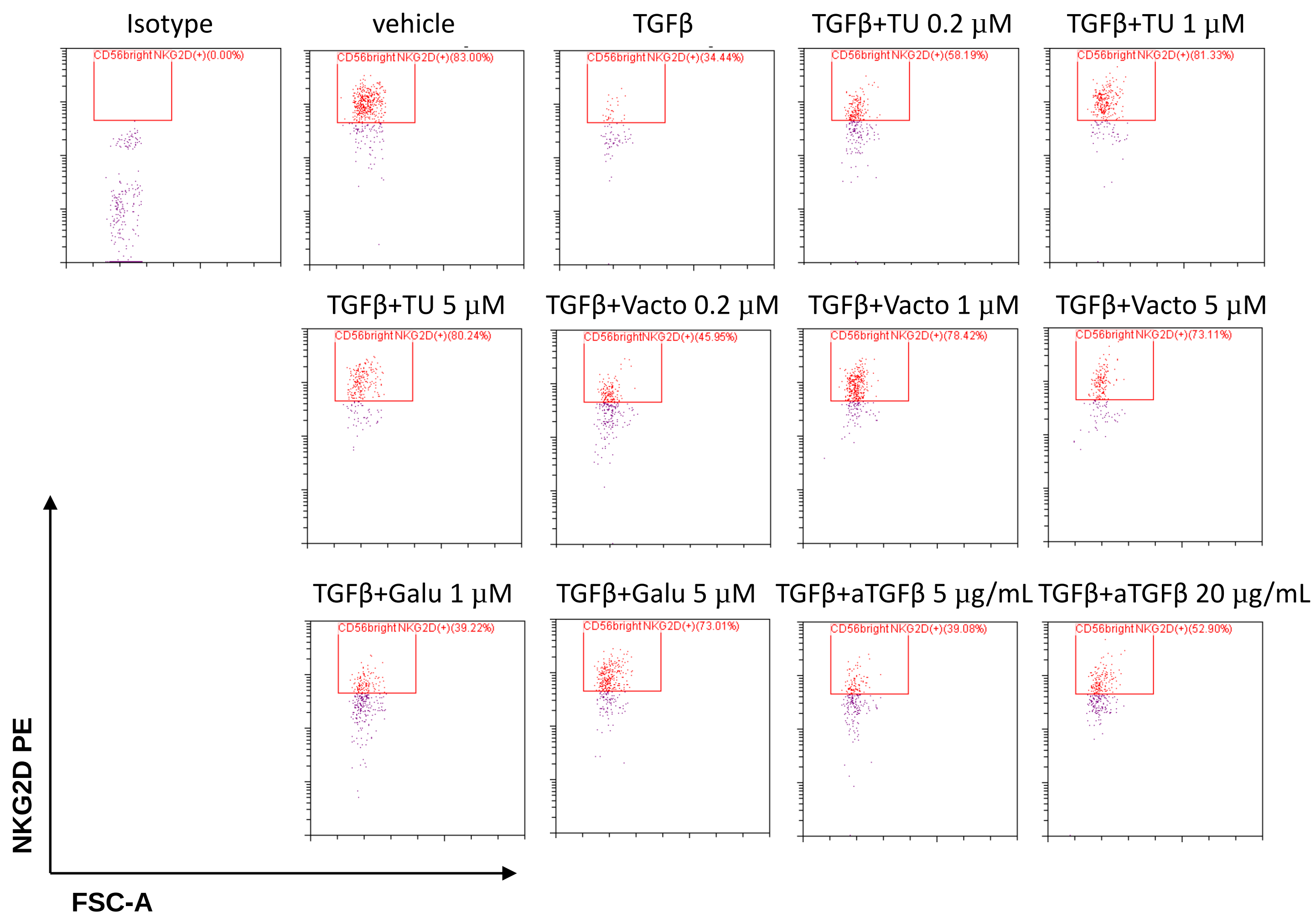

Group	Cell (%)	Relative Cell (%)
Isotype	0	
vehicle	83.00	100.0
TGFβ	34.44	41.5
TGFβ+TU2218 0.2 μM	58.19	70.1
TGFβ+TU2218 1 μM	81.33	98.0
TGFβ+TU2218 5 μM	80.24	96.7
TGFβ+Vactosertib 0.2 μM	45.95	55.4
TGFβ+Vactosertib 1 μM	78.42	94.5
TGFβ+Vactosertib 5 μM	73.11	88.1
TGFβ+Galunisertib 1 μM	39.22	47.3
TGFβ+Galunisertib 5 μM	73.01	88.0
TGFβ+anti-TGFβ 5 μg/mL	39.08	47.1
TGFβ+anti-TGFβ 20 μg/mL	52.90	63.7

Abbreviation; TU; TU2218, Vacto; Vactosertib, Galu; Galunisertib, aTGFβ; anti-TGFβ

Supplementary Figure S1. Gating strategy and FCM graph for Figure 2

(d) Gating strategy of live cell of NK92mi for Figure 2d (left)

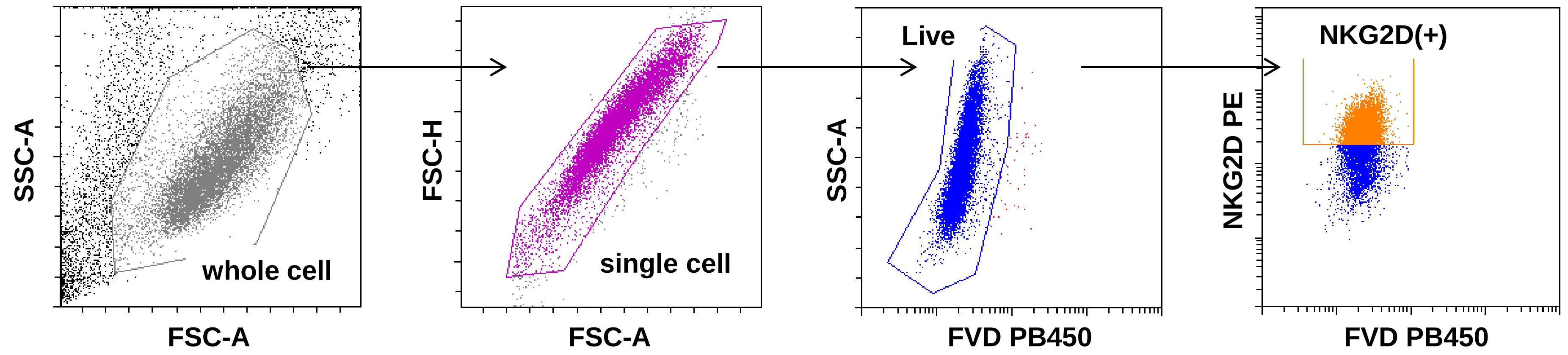

(e) Representative FCM graph for Figure 2d (left)

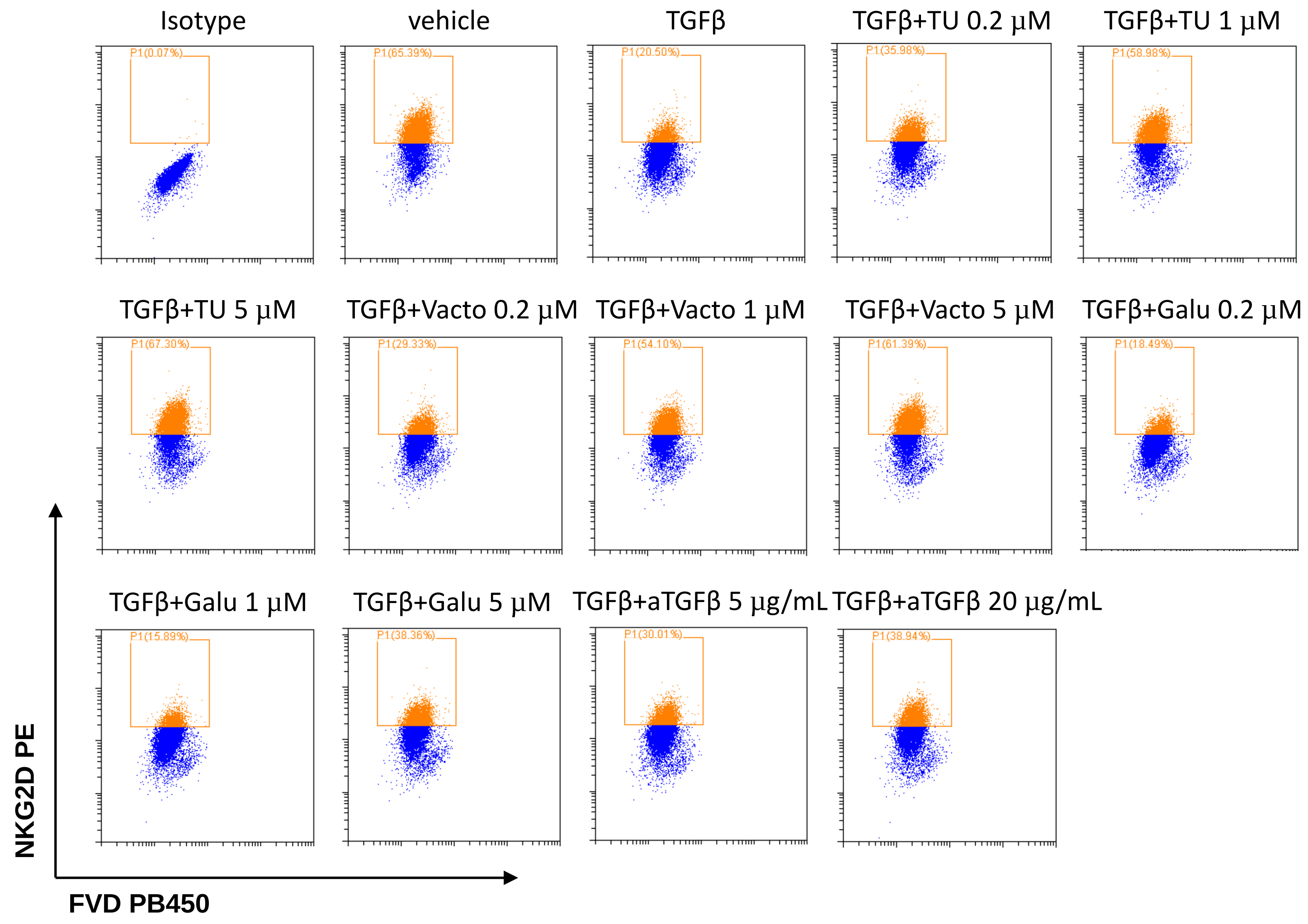

Group	Cell(%)	Relative Cell (%)
Isotype	0.07	-
vehicle	65.39	100.0
TGFβ	20.50	31.4
TGFβ+TU2218 0.2 μM	35.98	55.0
TGFβ+TU2218 1 μM	58.98	90.2
TGFβ+TU2218 5 μM	67.30	102.9
TGFβ+Vactosertib 0.2 μM	29.33	44.9
TGFβ+Vactosertib 1 μM	54.10	82.7
TGFβ+Vactosertib 5 μM	61.39	93.9
TGFβ+Galunisertib 0.2 μM	18.49	28.3
TGFβ+Galunisertib 1 μM	15.89	24.3
TGFβ+Galunisertib 5 μM	38.36	58.7
TGFβ+anti-TGFβ 5 μg/mL	30.01	45.9
TGFβ+anti-TGFβ 20 μg/mL	38.94	59.6

Abbreviation; TU; TU2218, Vacto; Vactosertib, Galu; Galunisertib, aTGFβ; anti-TGFβ

Supplementary Figure S1. Gating strategy and FCM graph for Figure 2

(f) Gating strategy of K562 (CFSE(+)) for Figure 2d (right)

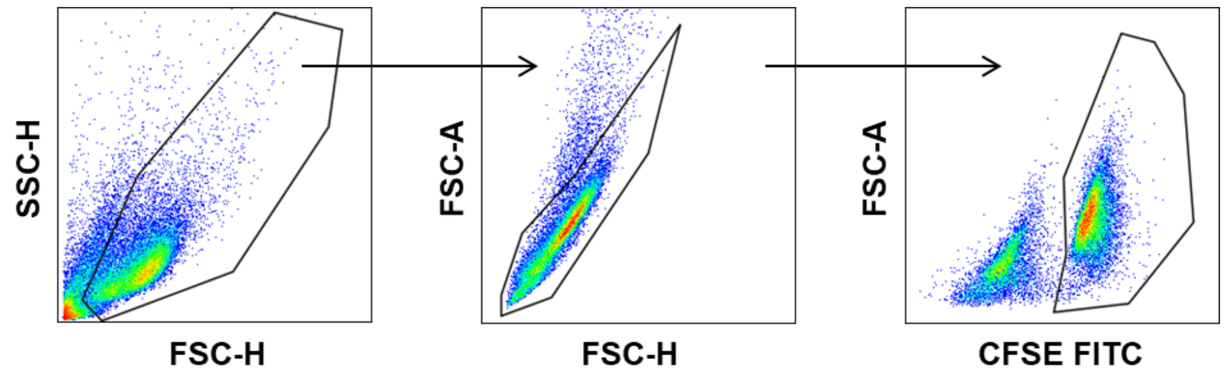

(g) Representative FCM graph for Figure 2d (right)

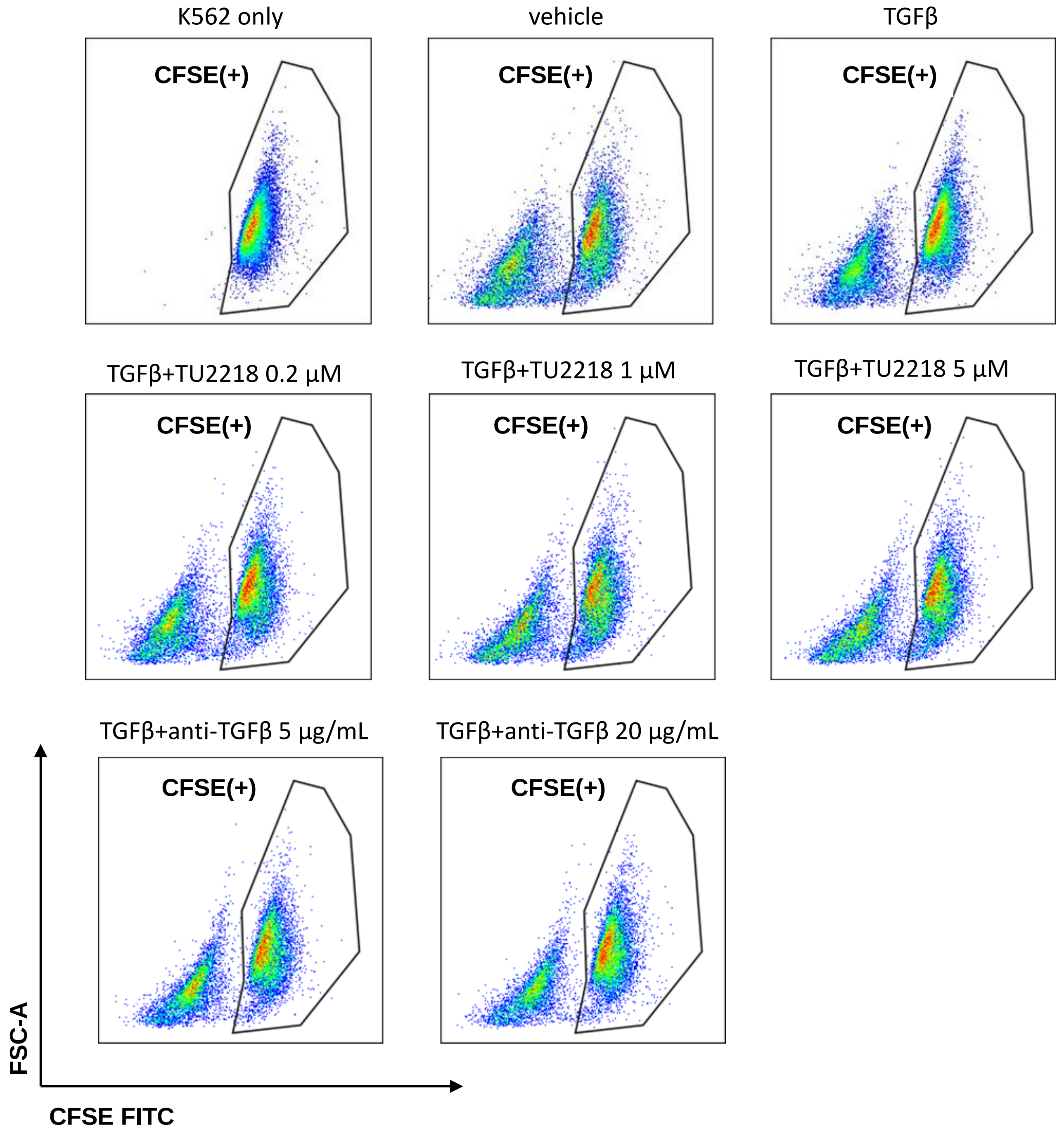

Group	CFSE(+) number	Cytotoxicity(%)
K562 only	18475	0.0
vehicle	8545	53.7
TGFβ	13151	28.8
TGFβ+TU2218 0.2 μM	9825	46.8
TGFβ+TU2218 1 μM	8094	56.2
TGFβ+TU2218 5 μM	8780	52.5
TGFβ+anti-TGFβ 5 μg/mL	9978	46.0
TGFβ+anti-TGFβ 20 μg/mL	10286	44.3

Supplementary Figure S2. Gating strategy and FCM graph for Figure 5

(a) Gating strategy of Jurkat T cell (CFSE(+)) for Figure 5b, 5c

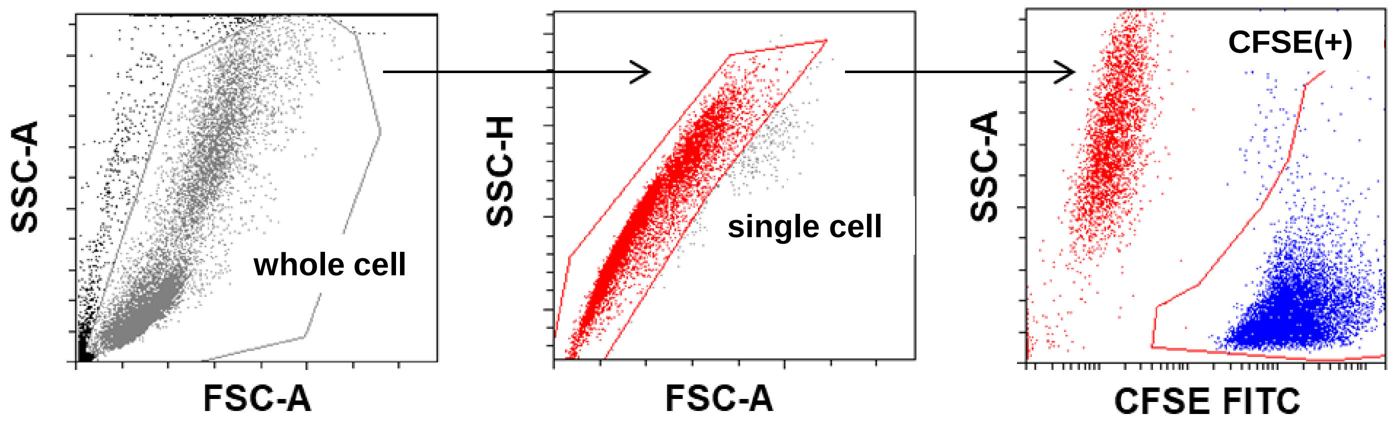

(b) Representative FCM graph for Figure 5b (upper)

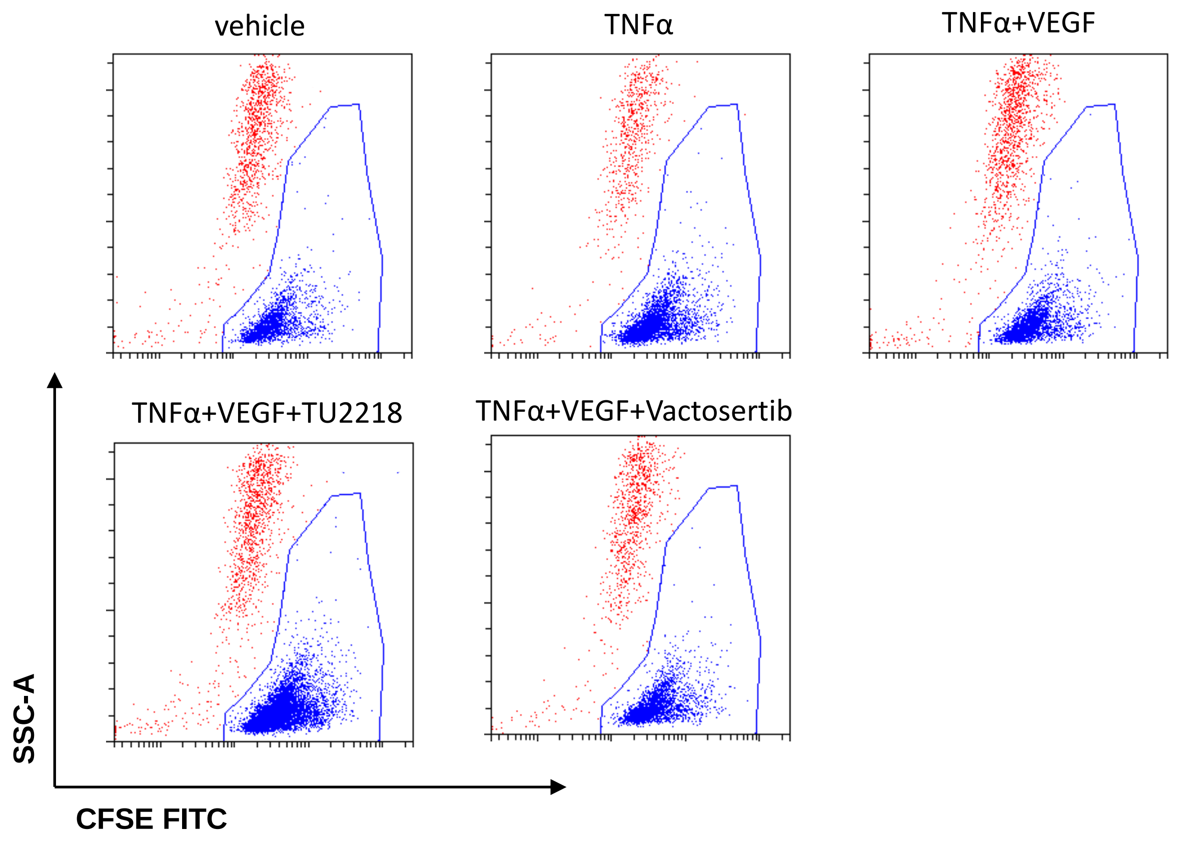

Group	Jurkat cell number
vehicle	2079
TNFα	3614
TNFα+VEGF	2477
TNFα+VEGF+TU2218	7282
TNFα+VEGF+Vactosertib	2782

(c) Representative FCM graph for Figure 5b (bottom)

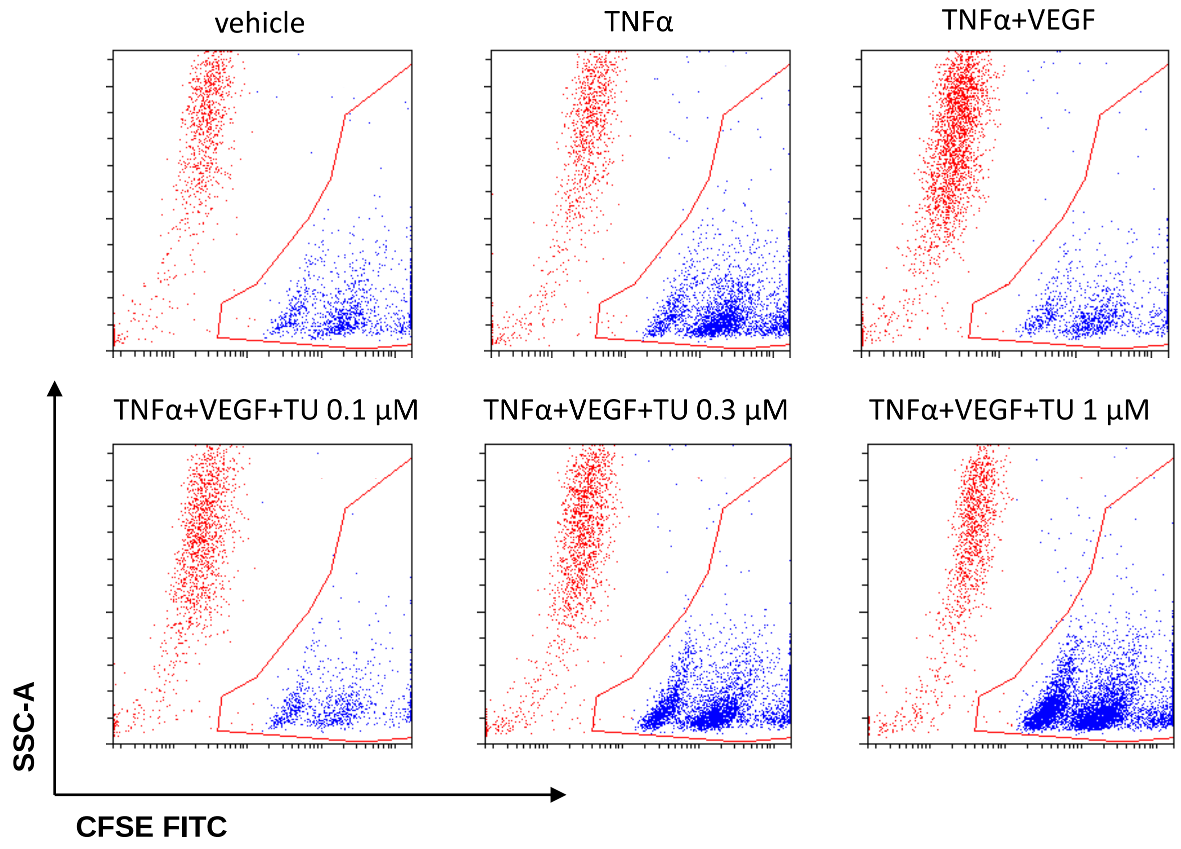

Group	Jurkat cell number
vehicle	1271
TNFα	3035
TNFα+VEGF	1189
TNFα+VEGF+TU2218 0.1 μM	921
TNFα+VEGF+TU2218 0.3 μM	3426
TNFα+VEGF+TU2218 1 μM	5790

Abbreviation; TU; TU2218

Supplementary Figure S2. Gating strategy and FCM graph for Figure 5

(d) Representative FCM graph for Figure 5c (left)

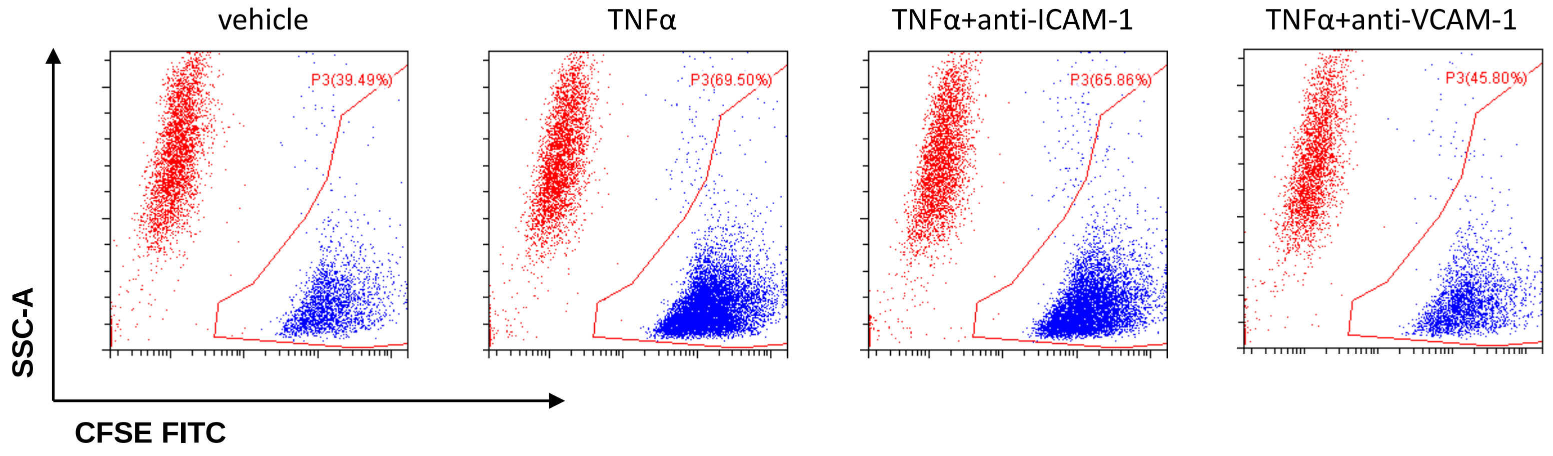

Group	Jurkat cell number	Number of Jurkat cell (%)
vehicle	2266	27.7
TNFα	8173	100.0
TNFα+anti-ICAM-1	6909	84.5
TNFα+anti-VCAM-1	2461	30.1

(e) Representative FCM graph for Figure 5c (right)

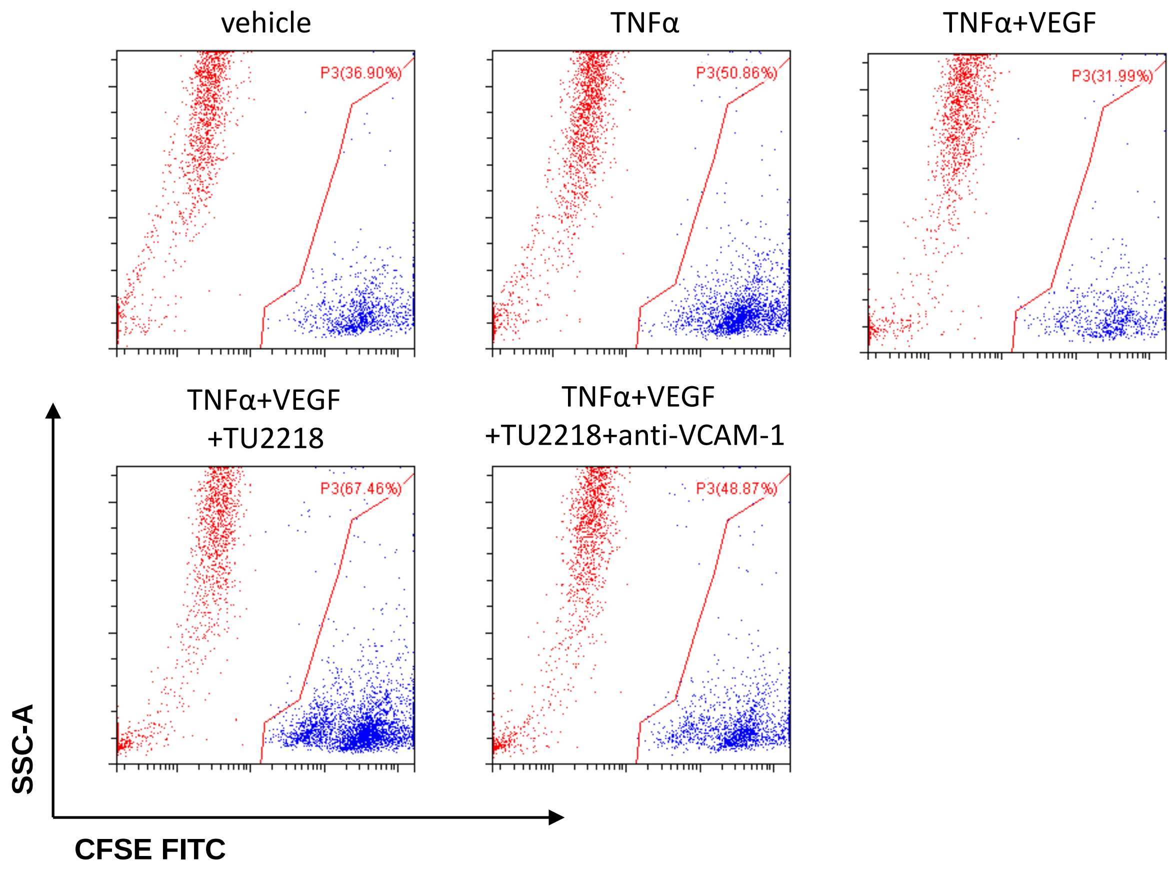

Group	Jurkat cell number	Number of Jurkat cell (%)
vehicle	1037	50.4
TNFα	2059	100.0
TNFα+VEGF	744	36.1
TNFα+VEGF+TU2218	3002	145.8
TNFα+VEGF+TU2218+anti-VCAM-1	1487	72.2

Supplementary Figure S3. Gating strategy for Figure 8

(a) Gating strategy of T_{EM} of CD4+ and T_{EM} of CD8+ in spleen tissue for Figure 8c

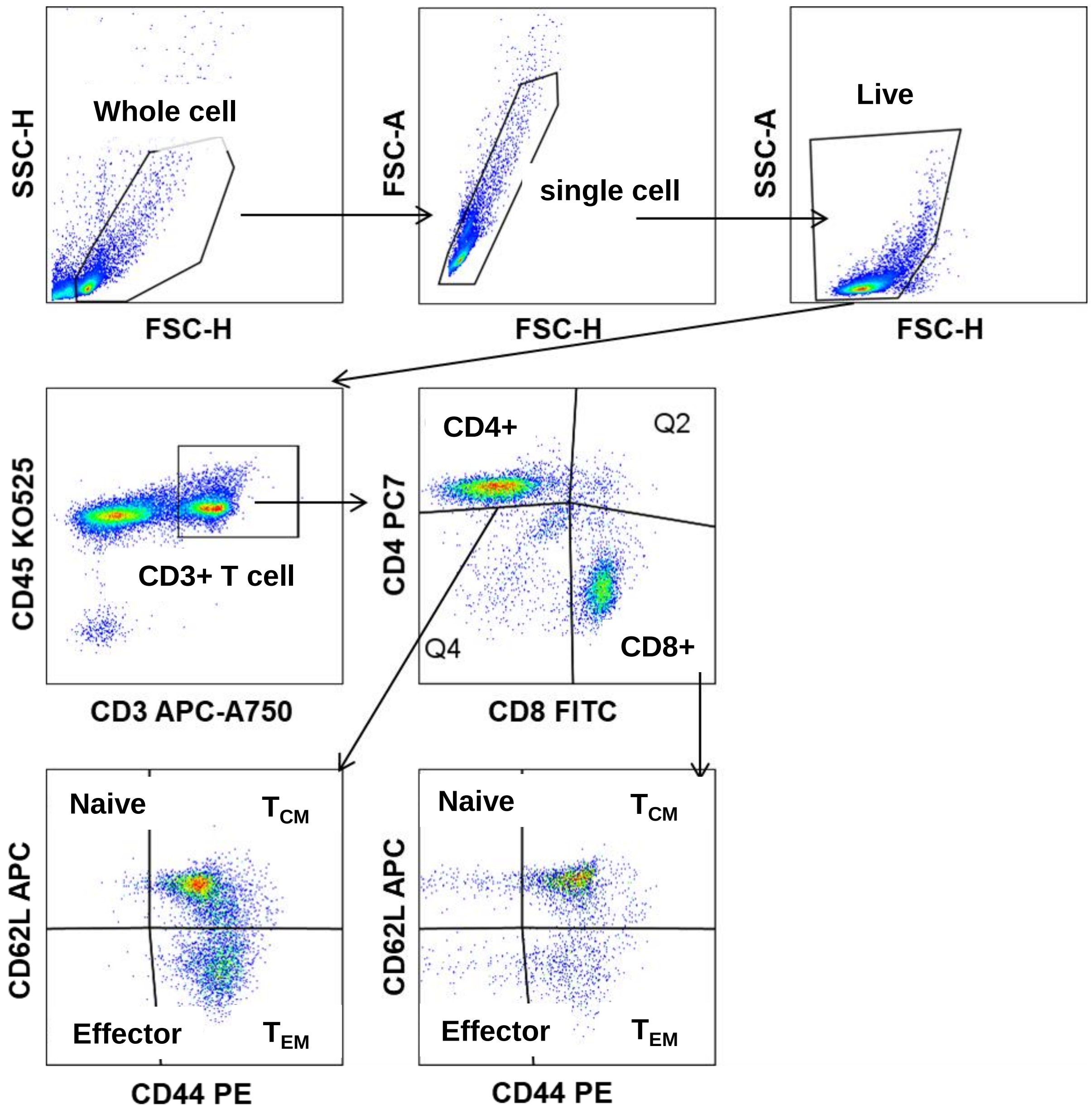

(b) Representative FCM graph for Figure 8c (left, CD4+)

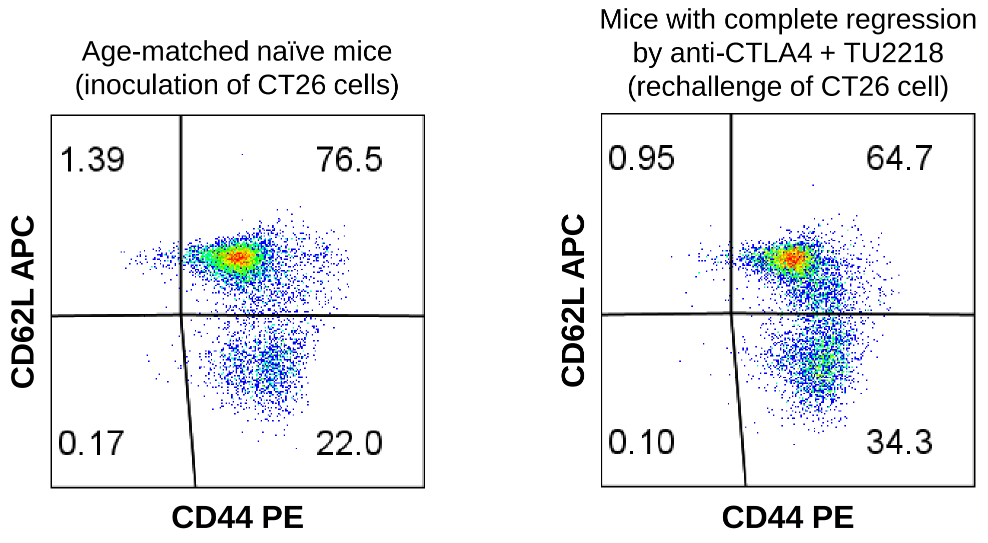

(c) Representative FCM graph for Figure 8c (right, CD8+)

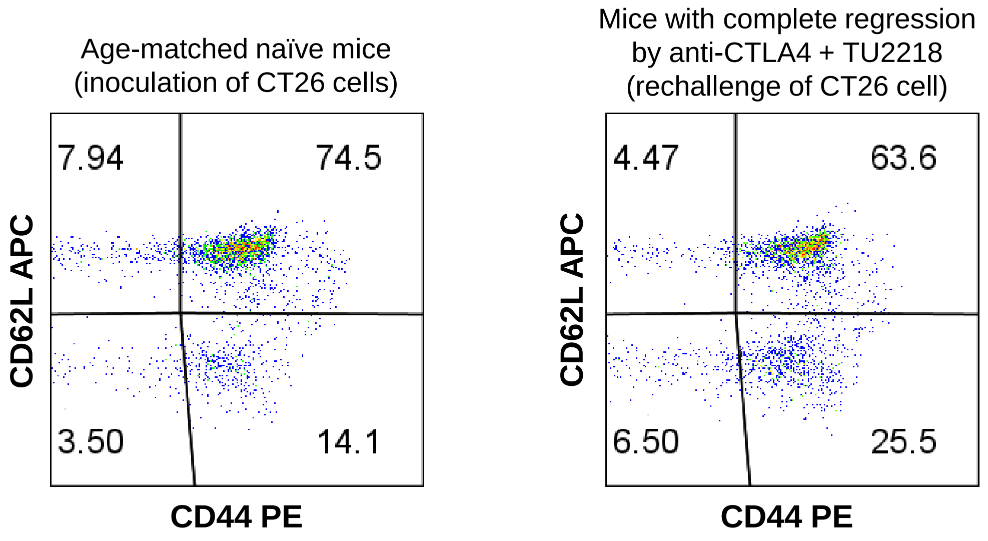

Supplementary Table S1. List of gene ontology (Biological process) of Up-regulated gene (p-value<0.05)

Term	P-Value
inflammatory response	1.2E-07
angiogenesis	1.3E-07
cellular response to lipopolysaccharide	0.000035
cell adhesion	0.00008
Ras protein signal transduction	0.00024
cellular response to beta-amyloid	0.00045
response to muramyl dipeptide	0.00067
positive regulation of vascular smooth muscle cell proliferation	0.00068
heterophilic cell-cell adhesion via plasma membrane cell adhesion molecules	0.00072
signal transduction	0.00076
immune response	0.0011
response to lipopolysaccharide	0.0012
positive regulation of gene expression	0.0013
response to hypoxia	0.0019
nervous system development	0.0023
cell-cell adhesion	0.0037
apoptotic process	0.0037
leukocyte cell-cell adhesion	0.0048
positive regulation of ERK1 and ERK2 cascade	0.0061
Notch signaling pathway	0.0067
cellular response to tumor necrosis factor	0.01
ERK1 and ERK2 cascade	0.014
positive regulation of smooth muscle cell proliferation	0.015
membrane to membrane docking	0.016
positive regulation of synapse assembly	0.016
regulation of cell proliferation	0.018
response to nematode	0.022
axon guidance	0.024
cellular response to mechanical stimulus	0.025
positive regulation of osteoblast differentiation	0.025
positive regulation of synaptic plasticity	0.026
cyclooxygenase pathway	0.029
chronic inflammatory response	0.032
T-helper 1 cell differentiation	0.035
response to molecule of bacterial origin	0.035
Notch signaling involved in heart development	0.035
wound healing	0.037
defense response to virus	0.04
cardiac septum morphogenesis	0.041
embryonic morphogenesis	0.041
positive regulation of transcription from RNA polymerase II promoter	0.043
positive regulation of tumor necrosis factor production	0.046
heart trabecula formation	0.048
auditory receptor cell differentiation	0.048
positive regulation of inflammatory response	0.049

Supplementary Table S2. List of gene ontology (Biological process) of Down-regulated gene (p-value<0.05)

Term	P-Value
mitotic spindle organization	0.000025
chromosome segregation	0.00011
cell division	0.0009
mitotic cytokinesis	0.001
mitotic cell cycle	0.001
mitotic chromosome condensation	0.0013
mitotic spindle assembly checkpoint	0.004
establishment of mitotic spindle orientation	0.005
mitotic sister chromatid segregation	0.0053
animal organ regeneration	0.0053
animal organ development	0.0059
positive regulation of ovulation	0.0088
regulation of synaptic plasticity	0.011
female meiosis chromosome segregation	0.012
mitotic chromosome movement towards spindle pole	0.012
G2/M transition of mitotic cell cycle	0.013
peptidyl-serine phosphorylation	0.017
cellular response to luteinizing hormone stimulus	0.017
hematopoietic progenitor cell differentiation	0.023
blood coagulation	0.029
Ras protein signal transduction	0.031
protein localization to kinetochore	0.035
attachment of mitotic spindle microtubules to kinetochore	0.035
cellular response to follicle-stimulating hormone stimulus	0.035
proteolysis	0.035
negative regulation of fibrinolysis	0.038
metaphase plate congression	0.04
cellular response to fluid shear stress	0.04
kinetochore assembly	0.043
regulation of NMDA receptor activity	0.046
regulation of small GTPase mediated signal transduction	0.048

Supplementary Table S3. The primer sequence of qRT-PCR

Gene	Type	Primer sequences
Up-regulated gene		
VCAM1	Forward	CTG TCA CTC GAG ATC TTG AGG
	Reverse	CCT GCA GTG CCC ATT ATG A
ICAM1	Forward	CCC TTG ACC GGC TGG AGA TT
	Reverse	CTG GGG GCA ACA TTG ACA TAA AGT G
COL8A1	Forward	GCC ACC TCA AAT TCC TCC TCA
	Reverse	CTT GTT CCC CTC GTA AAC TGG
IL32	Forward	AGG ACG TGG ACA GGT GAT GTC
	Reverse	GTC TCC AGG TAG CCC TCT TTG A
PDLIM1	Forward	AAT GTG GCA CTG GGA TTG TT
	Reverse	GAA ATG GCC CTT CTG TTT CA
CDON	Forward	GTT AAC TGC CGA AAT TGT CGA AA
	Reverse	TGC TAC CAC AGG GAC CAC AG
CLDN11	Forward	CGT GGG TGG CTG TGT CAT C
	Reverse	GAG CCC GCA GTG TAG TAG AAA C
CDH6	Forward	TAT CAG ACC CCG ACC ATA TT
	Reverse	GAC CAT AAA CTT CCG GCT T
NRCAM	Forward	AGT GTG TGA GTC TCA GCA GG
	Reverse	TGT TGG GTG ATG GTT GGA GG
BMX	Forward	CAGATTGTCTATAAAGATGGGC
	Reverse	TGTAATGCTTTCAACCACTG
Down-regulated genes		
NCAPH	Forward	GCC AGC CAC AAT GAA TAA
	Reverse	TGC AGA TCA AAG ACC CTC
PLK1	Forward	GCC CCT CAC AGT CCT CAA TA
	Reverse	TAC CCA AGG CCG TAC TTG TC
SPRY1	Forward	GCC TTC TTT GGA TAG CCG TCA G
	Reverse	TCA TTG CTG CCT CTT ATG GCC
CENPE	Forward	GAT TCT GCC ATA CAA GGC TAC AA
	Reverse	TGC CCT GGG TAT AAC TCC CAA
PBK	Forward	CCA AAC ATT GTT GGT TAT CGT GC
	Reverse	GGC TGG CTT TAT ATC GTT CTT CT
DLGAP5	Forward	AAG TGG GTC GTT ATA GAC CTG A
	Reverse	TGC TCG AAC ATC ACT CTC GTT AT
KIF20A	Forward	TGC TGT CCG ATG ACG ATG TC
	Reverse	AGG TTC TTG CGT ACC ACA GAC
CDK1	Forward	TGA GGT AGT AAC ACT CTG GTA
	Reverse	ATG CTA GGC TTC CTG GTT
CCNA2	Forward	CGC TGG CGG TAC TGA AGT C
	Reverse	GAG GAA CGG TGA CAT GCT CAT
NDC80	Forward	CCT CTC CAT GCA GGA GTT AAG A
	Reverse	GGT CTC GGG TCC TTG ATT TTC T
TPX2	Forward	ATG GAA CTG GAG GGC TTT TTC
	Reverse	TGT TGT CAA CTG GTT TCA AAG GT
NUSAP1	Forward	AGC CCA TCA ATA AGG GAG GG
	Reverse	ACC TGA CAC CCG TTT TAG CTG
ALNL	Forward	CAA GAT GTA TCC AAT GAC T
	Reverse	TGA CTG AAG AAT GAA TGT T
TOP2A	Forward	ACC ATT GCA GCC TGT AAA TGA
	Reverse	GGG CGG AGC AAA ATA TGT TCC
House-keeping gene		
GAPDH	Forward	ACC CAG AAG ACT GTG GAT GG
	Reverse	TTC TAG ACG GCA GGT CAG GT
